# Far-Red Light-Induced *Azolla filiculoides* Symbiosis Sexual Reproduction: Responsive Transcripts of Symbiont *Nostoc azollae* Encode Transporters Whilst Those of the Fern Relate to the Angiosperm Floral Transition

**DOI:** 10.3389/fpls.2021.693039

**Published:** 2021-08-11

**Authors:** Laura W. Dijkhuizen, Badraldin Ebrahim Sayed Tabatabaei, Paul Brouwer, Niels Rijken, Valerie A. Buijs, Erbil Güngör, Henriette Schluepmann

**Affiliations:** ^1^Laboratory of Molecular Plant Physiology, Department of Biology, Utrecht University, Utrecht, Netherlands; ^2^Department of Biotechnology, College of Agriculture, Isfahan University of Technology, Isfahan, Iran

**Keywords:** *Azolla*, *Nostoc*, dual RNA-sequencing, sexual reproduction, microRNA, MIKC and GAMYB transcription factors

## Abstract

Water ferns of the genus *Azolla* and the filamentous cyanobacteria *Nostoc azollae* constitute a model symbiosis that enabled the colonization of the water surface with traits highly desirable for the development of more sustainable crops: their floating mats capture CO_2_ and fix N_2_ at high rates using light energy. Their mode of sexual reproduction is heterosporous. The regulation of the transition from the vegetative phase to the spore forming phase in ferns is largely unknown, yet a prerequisite for *Azolla* domestication, and of particular interest as ferns represent the sister lineage of seed plants. Sporocarps induced with far red light could be crossed so as to verify species attribution of strains from the Netherlands but not of the strain from the Anzali lagoon in Iran; the latter strain was assigned to a novel species cluster from South America. Red-dominated light suppresses the formation of dissemination stages in both gametophyte- and sporophyte-dominated lineages of plants, the response likely is a convergent ecological strategy to open fields. FR-responsive transcripts included those from MIKC^C^ homologues of CMADS1 and miR319-controlled GAMYB transcription factors in the fern, transporters in *N. azollae*, and ycf2 in chloroplasts. Loci of conserved microRNA (miRNA) in the fern lineage included miR172, yet FR only induced miR529 and miR535, and reduced miR319 and miR159. Phylogenomic analyses of MIKC^C^ TFs suggested that the control of flowering and flower organ specification may have originated from the diploid to haploid phase transition in the homosporous common ancestor of ferns and seed plants.

## Introduction

Ferns from the genus *Azolla* thrive floating on freshwater. They require no nitrogen while growing even at the highest rate due to an unusual symbiosis with the cyanobacterium *Nostoc azollae*, which fixes dinitrogen using light energy (Brouwer et al., 2017). They constitute model plant symbioses allowing to study important traits, for example, a highly efficient N_2_ fixation imparted by a microbial consortium, the buildup of substrate mats capable of massive CO_2_ draw-down (Speelman et al., 2009), and a set of rare adaptations to the aquatic floating lifestyle in flood-prone regions. Previously used as biofertilizer, they now are considered as crops for the sustainable high-yield production of plant protein in subsiding delta regions but they have never been domesticated (Brouwer et al., 2014, 2018). What controls their sexual reproduction is completely unknown yet central to containment, propagation, breeding schemes, or the establishment of biodiversity and germplasm banks. Insight into the mechanisms controlling sexual reproduction in ferns is of particular interest as it will further uncover how these mechanisms evolved from a common homosporous ancestor because ferns and seed plants are from sister lineages.

*Azolla* spp. (hereafter Azolla) are heterosporous with diploid sporophytes generating two types of sori, mostly called sporocarps (Coulter et al., 1910; Nagalingum et al., 2006). The Azolla haploid phase, the gametophyte, is not free-living as in the case of homosporous ferns, but contained inside the sporocarp. The Azolla gametophyte develops inside the sporocarp after the later detaches from the sporophyte, unlike gametophytes from seed plants. Therefore, fertilization is not guided by structures, such as flowers in angiosperms, on the sporophyte. Azolla sporocarps typically detach when they mature and sink to the sediment of ditches; they constitute resting stages with nutrient reserves that survive drying or freezing and, thus, are keys to dissemination practices (Brouwer et al., 2014).

The developmental transition to sexual reproduction in the Azolla sporophyte begins when shoot apices of the sporophytes form initials of sporocarps (IS) in addition to initials of branches, leaves and roots. In homosporous ferns, IS corresponds to the onset of sori formation, of which the control is not well-studied; sori formation occurs mostly on leaves but fern leaves have indeterminate apical meristems as branches do (Cruz et al., 2020). In angiosperms, IS may be homologous to the transition of shoot apical meristems (SAM) to flower meristems (IF): the meristem is switched to make the branches with leaves and structures containing the sporangia, the “sporocarps,” whereby the making of sporocarps is ancestral to the making of leaves (Vasco et al., 2016). The gene expression in lycophytes and ferns of Class III HD-Zip (C3HDZ) transcription factors (TF) was observed in both leaves and sporangia even though ferns and lycophyte fossil records have shown that the lineages evolved leaves independently. Vasco et al. (2016), therefore, suggested that the common C3HDZ role in sporangium development was co-opted for leaf development when the leaf forms, the microphylls and megaphylls, evolved in lycophytes and ferns, respectively.

In the transition of SAM to IF of the model angiosperm *Arabidopsis thaliana* (Arabidopsis), the LEAFY TF complex induces floral homeotic genes (Sayou et al., 2016). LEAFY is conserved in plant lineages, but LEAFY does not promote the formation of reproductive structures in seed-free plants including ferns where, nonetheless, the protein was reported to maintain apical stem cell activity (Plackett et al., 2018). The emerging pattern, therefore, is that the LEAFY complex is not among those that were already in place to link exogenous cues to the transition of SAM to IS in the common ancestor of seed plants and ferns.

In many angiosperms, MIKC^C^-type MADS-box TF form the complexes that integrate endogenous and exogenous cues turning SAM into IF (Theißen et al., 2018). In Arabidopsis, for example, gibberellic acid-(GA-) dependent and photoperiod/temperature signaling is integrated by MIKC^C^ from the FLOWERING LOCUS C (FLC) and SHORT VEGETATIVE PHASE (SVP) clades that, as predicted by Vasco et al. (2016), are floral repressors. Consistent with separate transitions for inflorescence and flower meristems in Arabidopsis, the MIKC^C^ floral integrators act on another MIKC^C^, SUPPRESSOR OF CONSTANS 1 (AtSOC1), that in turn induces the expression of MIKC^C^ floral homeotic genes. Homeotic genes being potentially relevant for ferns specify ovules and stamens: these modules could be more ancient than those specifying carpels and sepals. The specification of ovules/stamens in angiosperms, however, is unlikely to be homologous to that of megasporocarps and microsporocarps in Azolla as heterospory evolved independently in ferns and angiosperms (Sessa and Der, 2016).

In addition to MIKC^C^ TF, microRNAs (miRs) are known to control phase transitions in angiosperms, including IF. The miR156/172 age pathway controls flowering: miRNA156 decreases with the age of plants, and thus also decreasing its repression of the targets *SQUAMOSA PROMOTER BINDING-LIKES* (*SPL*). In the annual Arabidopsis, SPL promote the expression of miR172 that targets another type of floral repressor, the APETALA 2 (AP2)/target of early activation tagged (TOE) TFs (Hyun et al., 2019). In the perennial *Arabis alpina*, the MIKC^C^ FLC homolog and miR156 regulate the expression of *SPL15*, thus integrating temperature- and age-dependent IF. Moreover, AP2/TOE and nodule inception (NIN) TF are known to mediate the repression of *AtSOC1* in Arabidopsis on high nitrate (Gras et al., 2018; Olas et al., 2019). The MIKC^C^ SOC1 is further known to control the levels of miR319 that inhibit TEOSINTE-LIKE1, CYCLOIDEA, and PROLIFERATING CELL FACTOR1 (TCP) protein functions in the FT–FD complex mediating the photoperiod control of IF (Lucero et al., 2017; Li et al., 2019). Inextricably, therefore, the transition to IF is linked to conserved microRNA (miRNA) modules in angiosperms, but we know little of miRNA loci in ferns (Berruezo et al., 2017; You et al., 2017).

**Figure 1:**
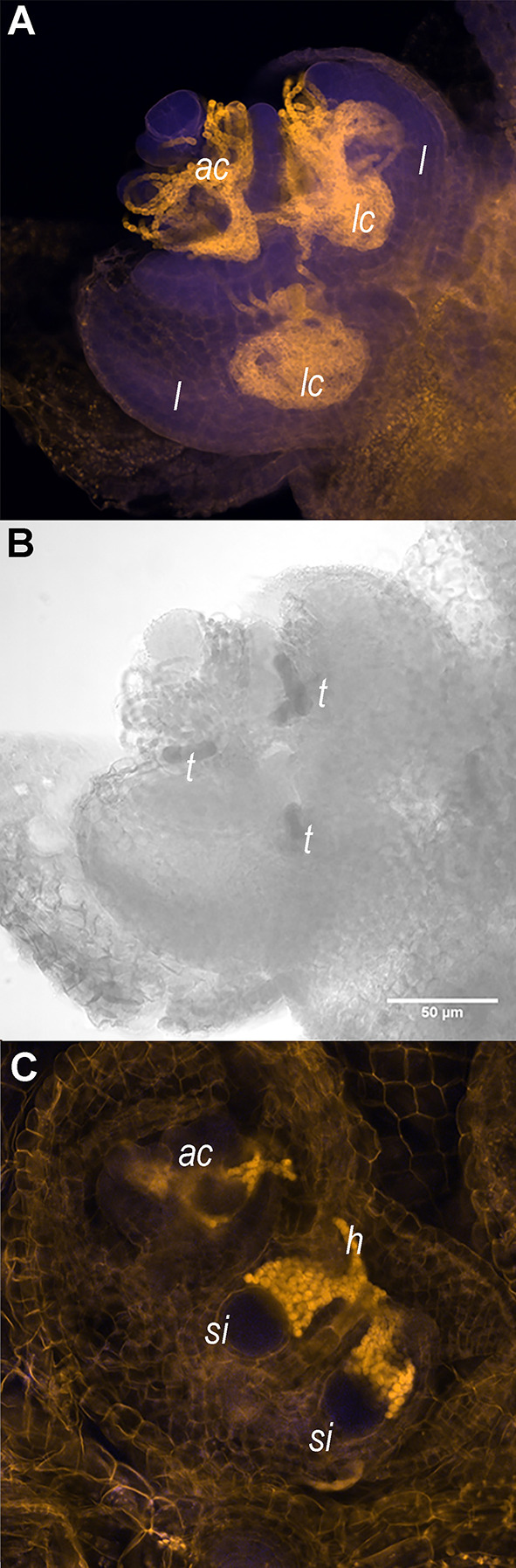
Shoot apices of *Azolla filiculoides* sporophytes contain the apical *Nostoc azollae* colony, trichomes, and sporocarp initials. **(A)** Shoot apex with apical *N. azollae* colony (*ac*). Outer leaves were removed for a better visualization of developing leaf lobes (*l*), leaf cavities (*lc*), and bacteria filaments. **(B)** The same shoot apex as in **(A)** with trichomes (*t*) revealed by transmitting light absorption surrounded by the apical colony and in areas destined to form leaf cavities (*lc*); Bar: 50 μm. **(C)** *N. azollae* hormogonia (*h*) invading the sporocarp initials (*si*). Sporophytes were stained with propidium iodide (Truernit et al., 2008), then the fluorescence was detected using 405 nm activation beam, and emission <560 nm filter for the yellow channel and 505–530 nm for the blue channel with the Confocal Laser Scanning microscope Zeiss LSM5 Pascal.

Systematic research on the Azolla symbiosis transition to IS is lacking: GA applications did not induce IS, neither did stress treatment (Kar et al., 2002). Moreover, fern host and symbiont development are tightly coordinated during the induction of sporocarps: the mobile filaments of *N. azollae* generated in the shoot apical colony move into the developing sporocarp initials in the shoot apex (Figure 1). The host SAM developmental transition, therefore, may be influenced by the activities of the *N. azollae* apical colony residing at the SAM (Figure 1A). Alternatively, the development of *N. azollae* may be controlled by the secretions from specialized host trichomes in the SAM, the developing leaf and sporocarp initials (Cohen et al., 2002; Perkins and Peters, 2006; Figures 1B,C). We expect an elaborate communication given that both genomes coevolved and that *N. azollae* is an obligate symbiont with an eroded genome (Ran et al., 2010; Dijkhuizen et al., 2018; Li et al., 2018). The transition to IS will, therefore, be best characterized by profiling the expression of the fern host and the symbiont simultaneously; this requires simultaneous profiling of eukaryotic and prokaryotic RNA [dual RNA-sequencing (RNA-seq)] and has not been established for cyanobacterial symbioses before.

Here, we identify the external cues that induce or inhibit the transition to IS in *Azolla filiculoides* under laboratory settings. We test the viability of the reproductive structures induced in different fern accessions by crosses, define the species attributions, and support the results obtained with phylogenetic analyses of the accessions. To reveal the endogenous mechanisms mediating the transition to IS, we profile transcript accumulation by dual RNA-seq comparing the sporophytes grown with and without far-red (FR) light at a time point when the reproductive structures are not yet visible. We assign the induced MIKC^C^ and MYB TF to the known clades using phylogenetic analyses. In addition, we profile small RNA (sRNA) from the symbiosis and then identify miRNAs and their targets in the fern lineage. Finally, we discover the regulatory modules of conserved miRNAs/targets responsive during the induction of IS in ferns.

## Materials and Methods

### Plant Materials, Growth Conditions, and Crosses

Ferns were collected from different locations in the Netherlands and grown in a liquid medium at a constant temperature of 22°C for long period (16 h light) as described earlier (Brouwer et al., 2017). Ferns from Spain and Iran were processed to DNA on site. GPS coordinates from the collection sites of the strains used in phylogenetic analyses are given in Supplementary Table 1. For sporocarp induction, ferns were first raised under a 16-h tube light (TL) period and then transferred for the induction under 16-h TL with FR light emitting diodes (LED; Phillips GrowLED peaking at 735 nm, Supplementary Figures 1A,B). Photosynthetic photon flux density varied in the range of 100–120 μmol m^−2^ s^−1^. Sporophytes were generally kept at the densities that covered the surface as this inhibits the growth of cyanobacteria and algae. Sporophytes were kept at set densities (with a fresh weight resetting once per week, Supplementary Figures 1C–E) and set red to FR light ratios (Supplementary Table 2) while testing sporocarp induction. A phenotypic change in mature sporophytes was photographed using a Nikon D300s with an AF Micro-Nikkor 60 mm f/2.8D objective or bellows on a rail to generate image reconstructions with the Helicon focus software. Megasporocarps were collected manually using tweezers, so were microsporocarps. To initiate crosses, massulae were first released by Dounce-homogenization of the microsporocarps, then the massulae were added to megasporocarps in distilled water (3–6 ml, in 3 cm diameter petri dish), shaking so as to obtain clumps. The crosses were kept at room temperature (about 18°C) under 16 h light conditions at TL of about 100 μmol m^−2^ s^−1^. As soon as sporelings were boyant enought to reach the water surface they were transferred to liquid medium (Brouwer et al., 2014).

### RNA Extraction From Sporophytes and Quantitative Real-Time PCR

RNA was extracted; DNAse was treated and then reverse-transcribed (Brouwer et al., 2014). Primers for qRT-PCR were for the references *AfTUBULIN* (AfTUBF: CCTCCGAAAACTCTCCTTCC; AfTUBR: GGGGGTGATCTAGCCAAAGT) and *AfADENINE PHOSPHORIBOSYLTRANSFERASE* (AfAPTF: TAGAGATGCATGTGGGTGCAGT; AfAPTR: AAAAGCGGTTTACCACCCAGTT), and for *AfSOC1* (AfSOCF: ATGGGATCGTAAGGCTTCAAAA; AfSOCR: AGCAGAGCACACAGGTCTCAAC). Quantitative real-time PCR (qRT-PCR) amplifications were from RNA of three biological replicate growth tubs, and significance was assessed by *t*-test with *p* < 0.05.

### Phylogenetic Analyses

#### Chloroplastic Marker Regions

DNA was extracted by using the extraction kit (EZNA SP Plant DNA, Omega Bio-tek, Inc., Norcross, GA, USA), PCR amplifications used *pfu* and *taq* polymerase mixed (1:4 units) with *pfu* bufffer containing MgSO_4_ (2 mM) and *taq*-cycling conditions (37 cycles of 30 s 94°C denaturation, 30 s 50–58°C annealing, 1 min 72°C amplification, and then 5 min 72°C) and used the primers trnG1F (GCGGGTATGGTTTAGTGGTAA), trnR22R (CTATCCATTAGACGATGGACG), trnLC (CGGAATGGTAGACGCTACG), and trnLF (ACTTGAACTGGTGACACGAG). Amplicons were purified (EZNA Cycle pure kit, Omega Bio-tek, Inc., Norcross, GA, USA) and then submitted for sequencing (Macrogen, Seoul, South Korea). Sequences were reconstructed and then added to a fasta file containing the cognate sequences from *Azolla* species sequenced previously (Madeira et al., 2016; Dijkhuizen et al., 2018). Sequences were aligned on MEGA 7 (Kumar et al., 2016) using Clustal W (Thompson et al., 1994). Gap opening and gap extension of 10 and 3, respectively, were used for trnL-trnF, and 10 and 4, respectively, were used for trnG-trnR. Maximum likelihood (ML) phylogenetic trees were calculated with alignment lengths of 902 and 852 bp for trnL-trnF and trnG-trnR, respectively. The trnL-trnF tree was constructed by the Tamura 3-parameter plus Gamma (T92 + I) model and the trnG-trnR tree using the Tamura 3-parameter plus Invariant (T92 + I) model. The gaps/missing data treatment was chosen as “partial deletion,” branch swap filter as “very weak,” and ML heuristic method as “Subtree–Pruning–Regrafting—Extensive (SPR level 5).” The reliability of branches was analyzed by using bootstrapping of 1,000 replicates; the obtained trees were visualized with an interactive tree of life (iTOL; Letunic and Bork, 2019).

#### Sequence Logo of ITS1 Intergenic Regions

The logo from the *A. filiculoides* internal transcribed spacer (ITS) was generated using WebLogo3 (Crooks et al., 2014) after extracting 516 sequences from the Azfi vs1 genome with *in silico* PCR (iPCRess, Slater and Birney, 2005) and the published reference sequence for *A. filiculoides* (Li et al., 2018). ITS1 sequences of other Azolla species (Dijkhuizen et al., 2018) were extracted by alignment to the *A. filiculoides* reference sequence, and the obtained Bam file was then used to generate the sequence logos of ITS regions from other Azolla including *Azolla nilotica* (section *Rhizosperma*), and *Azolla caroliniana 1, Azolla caroliniana 2, Azolla mexicana, Azolla microphylla*, and *Azolla rubra* (section Azolla).

#### MIKC^C^ and R2R3MYB Phylogenetic Trees

The automatically generated annotation of Azfi vs1 was first corrected in the Integrative Genomics Viewer (IGV; Thorvaldsdóttir et al., 2013) using reads from the dual RNA-seq experiment. Azolla fern sequences were then merged to those extracted from the genome browsers of each species and aligned with MAFFT L-INS-I or E-INS-I (Katoh et al., 2019), and then trimmed with trimAL (Capella-Gutiérrez et al., 2009). Phylogenetic inferences were computed with IQTREE (Nguyen et al., 2015) and its internal model fitter (Trifinopoulos et al., 2016). In case of MIKC^C^, the phylogeny obtained with MAFFT E-INS-I was used without trimming as draft phylogeny to guide alignment optimization with PRANK (Löytynoja, 2014); the resulting optimized alignment was then trimmed with trimAl and used for an inference of the final phylogeny with IQTREE. The resulting ML trees were visualized in iTOL (Letunic and Bork, 2019) with a minimum bootstrap support of 50%, and the sequences were color-coded based on their clade assignment (R2R3MYB) or on the phylogenetic placement (MIKC^C^).

### Reference Genomes Used to Determine Gene Counts From the Dual RNA-seq Reads

The assembly and annotation of the *A. filiculoides* accession Galgenwaard chloroplast and nuclear genomes were obtained from Li et al. (2018); the used strain was devoid of *N. azollae* and described previously by Brouwer et al. (2017). Because *A. filiculoides* species attribution was not entirely reliable, we first had to determine how different the genomes of *N. azollae* are within the *Azolla* genus and then determine what reference genome could be used for the *N. azollae* species inside the accession Galgenwaard. Metagenome assembled genomes (MAGs) from *N. azollae* in the *Azolla* species *A. nilotica, A. mexicana, A. microphylla, A. caroliniana 1, A. caroliniana 2, A. rubra*, and *A. filiculoides*. Galgenwaard strains were computed using the shotgun sequencing data of the accessions in Dijkhuizen et al. (2018): trimmed and quality assessed reads were filtered [BWA aligner (Li and Durbin, 2009)] against the fern and chloroplast genomes and then assembled into contiguous sequences (contigs) using SPAdes in a metagenomic mode (Nurk et al., 2017), and contigs assigned Streptophyta taxonomy with the addition of CAT (von Meijenfeldt et al., 2019) o the nucleus and chloroplast filter for another round of read filtering. The obtained reads were then assembled with SPAdes again, and then contigs are binned according to k-mer, GC, and vertical coverage of the reads on the contigs. Bins were further interactively polished in Anvi'o (Eren et al., 2015); the MAG sequence data are obtained under the accession PRJEB45214. Average nucleotide identity (ANI) was calculated with the dRep implementation of nucmer using the ANImf preset (Kurtz et al., 2004; Olm et al., 2017). Because MAGs were highly complete and ANI is >99% with *N. azollae* 708 from the *A. filiculoides* accession Stockholm (GCF_000196515.1), we therefore aligned reads and then derived read counts for the features encoded in this genome.

### Dual RNA-seq and Data Processing

*Azolla filiculoides* sporophytes were obtained from the sequenced accession Galgenwaard (Li et al., 2018). The ferns were maintained on liquid medium without nitrogen in long-day TL and then transferred to TL with and without FR LED for 7 days, on fresh liquid medium without nitrogen; a harvest of 2 h into the light period was done by snap freezing. All samples were grown separately and harvested as triplicate biological replicates. Total RNA was extracted by the Spectrum Plant Total RNA Kit (Sigma-Aldrich, St. Louis, MO, USA) using protocol B and then DNAse treated as described in Brouwer et al. (2017) and cleaned using the RNeasy MinElute Cleanup Kit (Qiagen, Hilden, Germany). Ribosomal RNA (rRNA) depletions were carried out as per the protocol using Ribo-Zero rRNA Removal Kit (Plant Leaf, Illumina, San Diego, CA, USA), and RNA was then cleaned again using the RNeasy MinElute Cleanup Kit (Qiagen, Hilden, Germany). Stranded libraries were then synthesized using the Ovation Complete Prokaryotic RNA-Seq Library Systems Kit (Nugen, Redwood City, CA, USA). The libraries thus obtained were characterized and quantified before sequencing using a single lane Hiseq4000 and 50 cycles PE reading (Illumina, San Diego, CA, USA).

The obtained reads were de-multiplexed and then trimmed and trimmomatic quality filtered (Bolger et al., 2014), yielding 13–60 million quality PE reads per sample; the data are under the accession number PRJEB45223. An alignment of the reads to the genome assembly and GFF vs1 of *A. filiculoides* (Azfi_vs1; Li et al., 2018) was made with STAR-aligner default settings (Dobin et al., 2013) and was combined with the STAR read count feature [PE read counts corresponding to the HTseq “Union” settings (Anders et al., 2015)]. Read count tables were fed into DESeq2 (Love et al., 2014) for an analysis of differentially accumulating transcripts.

STAR was also used to align the reads to the *N. azollae* and the chloroplast genomes, and the read counts were then extracted using STAR default parameters or VERSE (Zhu et al., 2016) with HTSeq setting “Union” comparing single and paired-end (PE) read counts on the polycistronic RNA. The three approaches yielded similarly ranked genes but the *P*-adjusted (*P*adj) statistics of differential expression in count tables from the forced single read counts were poor: only 20 *N. azollae* genes had *P*adj values smaller than 0.1 compared to up to 67 with the PE approaches, we therefore present the PE approach using STAR read counts only.

STAR alignment to the concatenated genomes of the *A. filiculoides* symbiosis using default settings yielded a large proportion of multi-mappers, which interfered with PE read counting leading to losses of as much as 30% of the reads uniquely mapped. The strategy would reduce force mapping, which is not necessary to extract differentially accumulating transcripts. In contrast, when mapping the sRNA reads mentioned below, we used alignments (allowing no mismatches) to the concatenated genomes and then recovered reads aligned to each genome using Samtools fasta from the indexed.bam files with the alignment data of the concatenated genomes.

### Small RNA Data Sequencing and Data Processing

Ferns were maintained on liquid medium without nitrogen with 16 h TL and then transferred to TL with and without FR LED for 7 days as described under Dual RNA-seq. In addition, sporophytes were grown on medium with or without 2 mM NH_4_NO_3_; ferns without cyanobacteria (Brouwer et al., 2017) were grown on medium with 2 mM NH_4_NO_3_ in TL with FR LED. All sporophytes following the conditions of 7 days into the treatment, 2 h into the light period, and snap frozen were harvested. The samples were harvested from triplicate growth experiments and thus represent triplicate biological replicates. sRNA was extracted as per the protocol using the mirPremier miRNA Isolation Kit (Sigma-Aldrich, St. Louis, MO, USA) and then DNAse treated (Brouwer et al., 2017) and cleaned using the Rneasy MinElute Cleanup Kit (Qiagen, Hilden, Germany) protocol for sRNA. Libraries of sRNA were then generated using the SeqMatic TailorMix miRNA Sample Preparation 12-reaction Kit (Version 2, SeqMatic, USA) as per the protocol. Libraries were characterized using an Agilent Technologies 2100 Bioanalyzer and quantified before sequencing using NextSeq500 and 75 b single read chemistry (Illumina, San Diego, CA, USA).

The reads thus obtained were de-multiplexed, trimmed, and trimmomatically quality filtered (the data are under the accession number PRJEB45223) and then aligned with STAR (Dobin et al., 2013) (set at maximal stringency and to output Bam files) to the concatenated fasta files of the genomes Azfi_vs1, its chloroplast, and *N. azollae* 0708. The resulting bam files were then split for each genome, and Samtools fasta (Li et al., 2009) was used to extract the reads aligned to each genome. The fastx collapser tool (http://hannonlab.cshl.edu/fastx_toolkit) was then used to extract the read counts for each identical sequence, with the sequence defining the name in the obtained lists. A data matrix was generated in bash and then exported to Excel or RStudio for further analyses.

To determine differential expression, the obtained lists were restricted to sRNA with at least 10 reads per sample joined in bash, and the tables thus obtained, first restricted to containing only reads of 20–22 nt sRNA (to minimize gel excision bias during sequencing library preparation), and then exported to R (RStudio Team, 2020). DESeq2 was then used to display variation and dispersion in the data and identify differentially accumulating sRNA using *P*adj values (Love et al., 2014).

### miRNA Discovery

The miRNA loci in *A. filiculoides* are analyzed, as shown in Supplementary Figure 2. Pre-miRNAs were predicted with miRDEEP-P2 (Kuang et al., 2019; multi-mapper minimum 100 and maximum loop length 500) and then extracted and subjected to secondary structure prediction using RandFold default parameters (Bonnet et al., 2004; Gruber et al., 2008). miRNA candidates containing less than four mismatches within the hairpin structure were viewed in IGV along with RNA expression data aligned to Azfi_vs1. The predicted miRNAs that were expressed in *A. filiculoides* were then compared in miRbase (Kozomara et al., 2019). The prediction of miRNA targets in Azfi_vs1 was made by using the intersect of both psRNATarget (Dai et al., 2018) and TargetFinder (Srivastava et al., 2014) with a cutoff of 5.0. The predicted targets were linked to the closest homolog using the Mercator annotation (Lohse et al., 2014) of Azfi_vs1 predicted proteins.

## Results

### FR Light and Canopy Density Induce, While Nitrogen in the Medium Represses, the Transition to Sexual Reproduction in *A. filiculoides*

**Figure 2:**
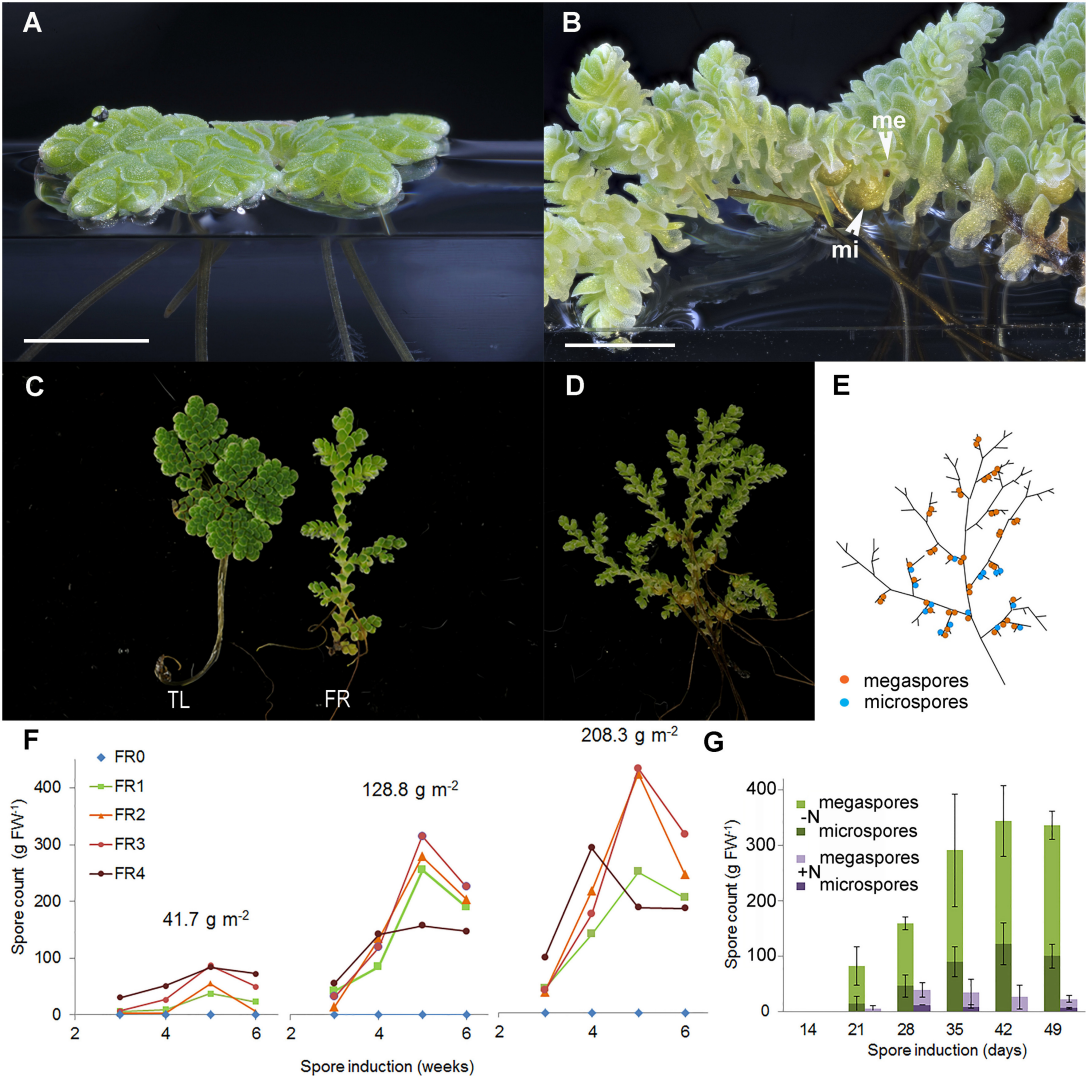
Far-red (FR) light and nitrogen affect the reproductive phase transition in *A. filiculoides*. **(A)** Vegetatively growing ferns. **(B)** Ferns in the reproductive phase with micro (mi) and megaspores (me). **(C)** Growth habit under tube light (TL) and TL with FR light emitting diodes (FR). **(D)** A mature sporophyte and **(E)** its schematic representation: a root is formed at each branch point, and sporocarp pairs are found close by on the branch side of the branch point but not at all branch points. **(F)** Sporulation frequencies at increasing plant density (41.7, 128.8, and 208.3 g m^−2^ dry weight) and with increasing red to FR light ratios (FR0–FR4). The red to FR ratios were 19.13 (FR0), 1.16 (FR1), 0.63 (FR2), 0.50 (FR3), and 0.31 (FR4); to obtain the FR4 ratio, the TL intensity was halved. Each data point represents the average of three independent continuous growth replicates. **(G)** Induction of sporulation without nitrogen (–N) or with 2 mM NH_4_NO_3_ (+N) in the medium, the data are averages from the three replicates with *SD*.

A young fern growing at low density typically spreads over the water surface (Figure 2A). In contrast, a fern that underwent the transition to reproductive development in dense canopies formed a three-dimensional web characteristic of Azolla mats (Figure 2B). Unlike in the glasshouse, *A. filiculoides* ferns never sporulated under TL (Supplementary Figure 1A); TL did not emit any photons in the FR range (FR, Supplementary Figure 1A). We, therefore, added FR during the entire 16 h light period (FR, Supplementary Figure 1B). Initially, this resulted in the elongation of the stem internodes of the fern (Figure 2C) and, after 2–3 weeks, in the formation of the sporocarps (Figure 2D). Sporocarps were positioned as pairs on the branch side of branch points and were predominantly arranged as a pair of megasporocarp and microsporocarp (Figure 2E).

To test the optimum light quality for sporocarp induction, decreasing ratios of red to FR were applied to sporophytes first raised in TL light. To test canopy density, light quality was also tested on the sporophytes maintained at three different densities (Supplementary Table 2, Supplementary Figures 1C–E). Far-red was required for sporocarp formation regardless of the density of the fern culture (Figure 2F). Increasing FR while keeping a constant photosynthetic active radiation (PAR) yielded more sporocarps per fern mass. The sporocarps per fern mass increased with the time of exposure to the FR until 5 weeks and then decreased, presumably because ripe sporocarps were detached. The sporocarps per fern mass, furthermore, increased with the density of the fern culture. Reducing the PAR at the highest FR intensity (Figure 2F, FR4) revealed that PAR-intensity limits the production of sporocarps at high FR.

Adding 2 mM ammonium nitrate to the medium drastically reduced the spore count when inducing sporocarp formation with FR (Figure 2G). Both ammonium and nitrate added individually inhibited sporocarp formation (data not shown); this inhibition explained why sporophytes without *N. azollae* kept on various nitrogen sources never sporulated.

The ratio of megasporocarp to microsporocarp was approximately equal over 9 weeks of FR induction (Supplementary Figure 1F). Additionally, the marker *SOC1-*like from *A. filiculoides* (Brouwer et al., 2014) was maximally induced when the sporocarps became visible 4 weeks after FR induction started (Supplementary Figure 1G).

### FR-Induced Sporocarps Are Viable, Which Permits Crossing for Breeding

We next wondered whether the sporocarps induced with FR were viable. We grew the following strains of *A. filiculoides* under FR light: *A. filiculoides* Galgenwaard (Li et al., 2018), six further accessions from the Netherlands, one from Gran Canaria (Spain), and another from the Anzali lagoon (Iran) (Supplementary Table 1). All accessions could be induced to sporulate under FR, except Anzali. Crosses of sporocarps obtained from within the Galgenwaard strain yielded viable and fertile offspring, so did crosses of sporocarps from the Hoogwoud accession (Table 1), suggesting that FR-induced sporocarps were generally viable. All sporulating accessions tested in crosses could be crossed, suggesting that the ferns from the Netherlands and Spain were *A. filiculoides* (Table 1). We were unable to induce sporulation in sporophytes from the Anzali lagoon, which had been reported as *A. filiculoides*, and therefore considered whether behavior under FR light and crosses predict phylogenetic attributions.

**Table 1:** Sporeling counts obtained from the random crosses of *Azolla filiculoides* strains collected in the Netherlands and Spain (Gran Canaria).

**Megaspores: Massulae:**	**Galgenwaard**	**Krommerijn**	**Hoogwoud**	**Gran Canaria**	**Den Bosch**	**Nijmegen**	**Nieuwebrug**	**Groningen**
Galgenwaard	>1,000	17		1		13		2
Krommerijn	1				16		2	
Hoogwoud			20					2
Gran Canaria^*^	17				>40	1		
Den Bosch		9		1				
Nijmegen		12		33			2	
Nieuwebrug		6	3			22		
Groningen			1				5	

* *Crosses involving megaspores from the Gran Canaria strain reproducibly germinated late, generally after 5 weeks instead of 2–3 weeks*.

### Phylogeny-Derived Species Attributions Are Consistent With Those Obtained From Crosses and Reveal That Anzali Ferns Were Not *A. filiculoides*

**Figure 3:**
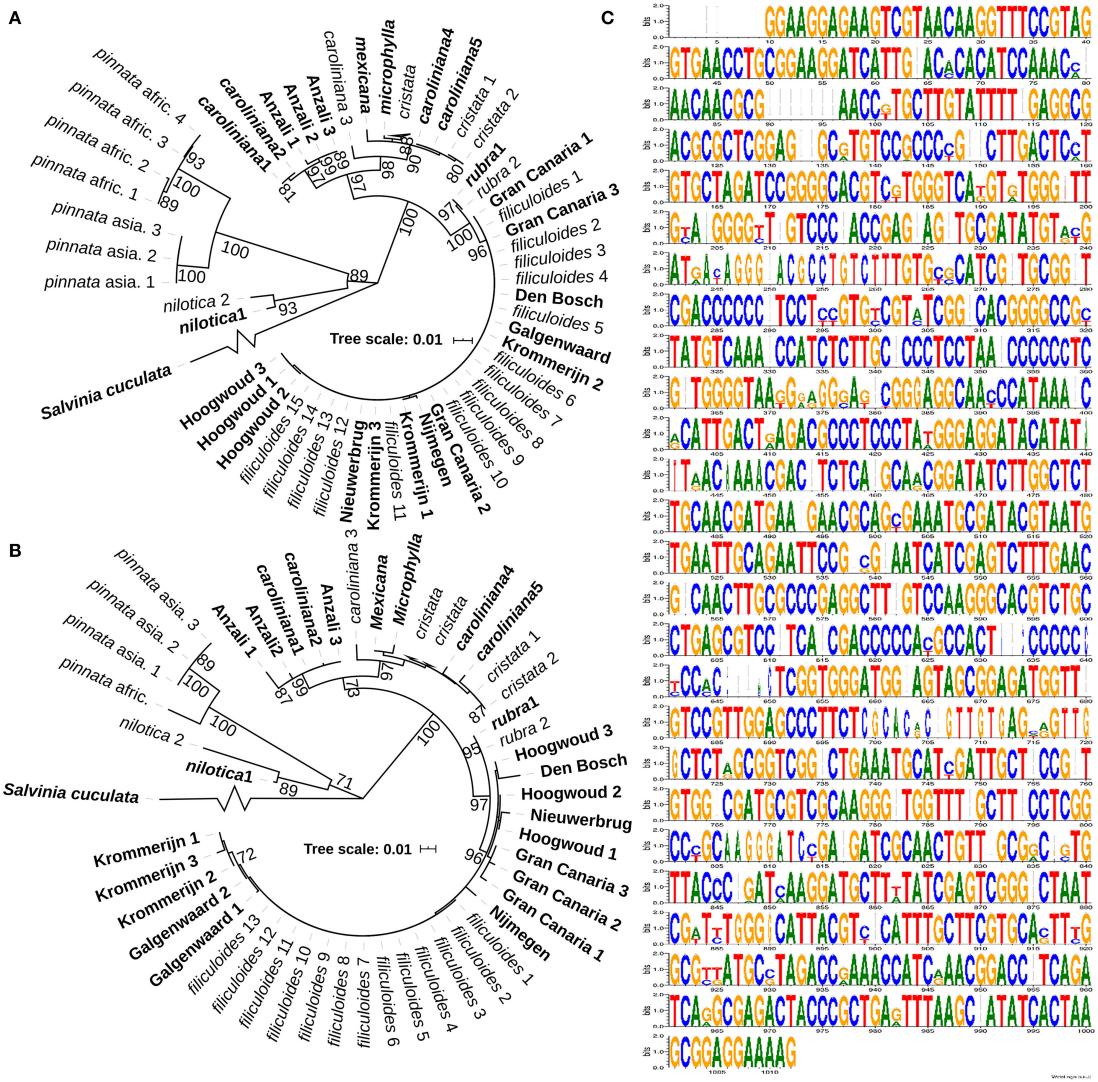
Chloroplast marker regions in strains from the Netherlands, Spain, and Iran, and the ribosomal RNA (rRNA) intergenic regions of the *A. filiculoides* genome. **(A)** Phylogenetic tree of chloroplast regions trnL-trnF. The maximum likelihood (ML) trees were bootstrapped 1,000x. All bootstrap values >0.70 are displayed. In bold: sequences from accessions in this study labeled after their collection site and sequences from the differing species extracted from genome shot-gun sequencing (Li et al., 2018). In regular type: sequences from the Madeira et al. (2016) study. **(B)** Phylogenetic tree of chloroplast regions trnG-trnR. **(C)** Sequence logo of all ITS1 gene regions in the *A. filiculoides* genome (Li et al., 2018). The gene regions were extracted and aligned (MEGA 7) and then used for *in silico* PCR. The results were visualized using WebLogo (vs. 2.8.2 Crooks et al., 2014): at each position of the ITS, the height of the stack indicates the sequence conservation, whereas the height of symbols within the stack indicates the relative frequency of each base.

To conduct research on the taxonomy of the accessions, intergenic regions of the chloroplast rRNA, used for phylogenetic studies previously (Madeira et al., 2016), were amplified and sequenced to compute phylogenetic relations. The regions trnL-trnF (Figure 3A) and trnG-trnR (Figure 3B) yielded similar trees confirming the close relationship of the *A. filiculoides* accessions from the Netherlands and Spain. The trees further revealed that the Anzali accession clustered along with two accessions from South America that do not cluster within the *A. caroliniana* group (Southern Brazil and Uruguay, Supplementary Table 1). The use of the nuclear ITS region for phylogeny reconstruction proved futile when amplifying the region on the *A. filiculoides* genome *in silico*: the *A. filiculoides* genome assembly contained over 500 copies of the ITS sequence, and the copies of ITS varied within the same genome (Figure 3C). Variations within any one genome were furthermore large when comparing between the genomes of *Azolla* species (Supplementary Figure 3A).

The dorsal side of the upper leaf lobes of the Anzali accession has two-cell, distinctly pointed, papillae unlike *A. filiculoides* that mostly, but not exclusively, have single-cell and rounded papillae (Supplementary Figure 3B). Consistent with its differing response to FR and its phylogenetic position based on the chloroplast sequences, the Anzali accession, we conclude, is not *A. filiculoides*.

### Dual RNA Sequencing After rRNA Depletion Allows the Profiling of Transcripts From Organelle, Prokaryotic Symbiont, and Fern Nucleus

We next wondered which molecular processes are altered during the azolla symbiosis transition to IS. Ferns maintained on TL were transferred to TL with and without FR for 1 week in triplicate cultures and then collected 2 h into the 16 h light period and snap frozen. Total RNA was then extracted, its plant rRNA was depleted, and stranded sequencing libraries were generated, which were depleted for rRNA from Gram-negative bacteria. Libraries were sequenced to obtain 12–60 million PE 50 bp long reads, quality filtered, and trimmed. To align the obtained reads, nuclear and chloroplast genomes with annotations for the *A. filiculoides* Galgenwaard accession were available (Li et al., 2018). In contrast, the genome of its *N. azollae* symbiont was not available as the long-read sequencing of the *A. filiculoides* Galgenwaard reference was done with the strain devoid of cyanobacteria (Li et al., 2018). In addition, we learned from above that species attributions can be misleading. We, therefore, decided to first determine how variable the genomes of the *N. azollae* are within the *A. filiculoides* species and the *Azolla* genus.

**Figure 4:**
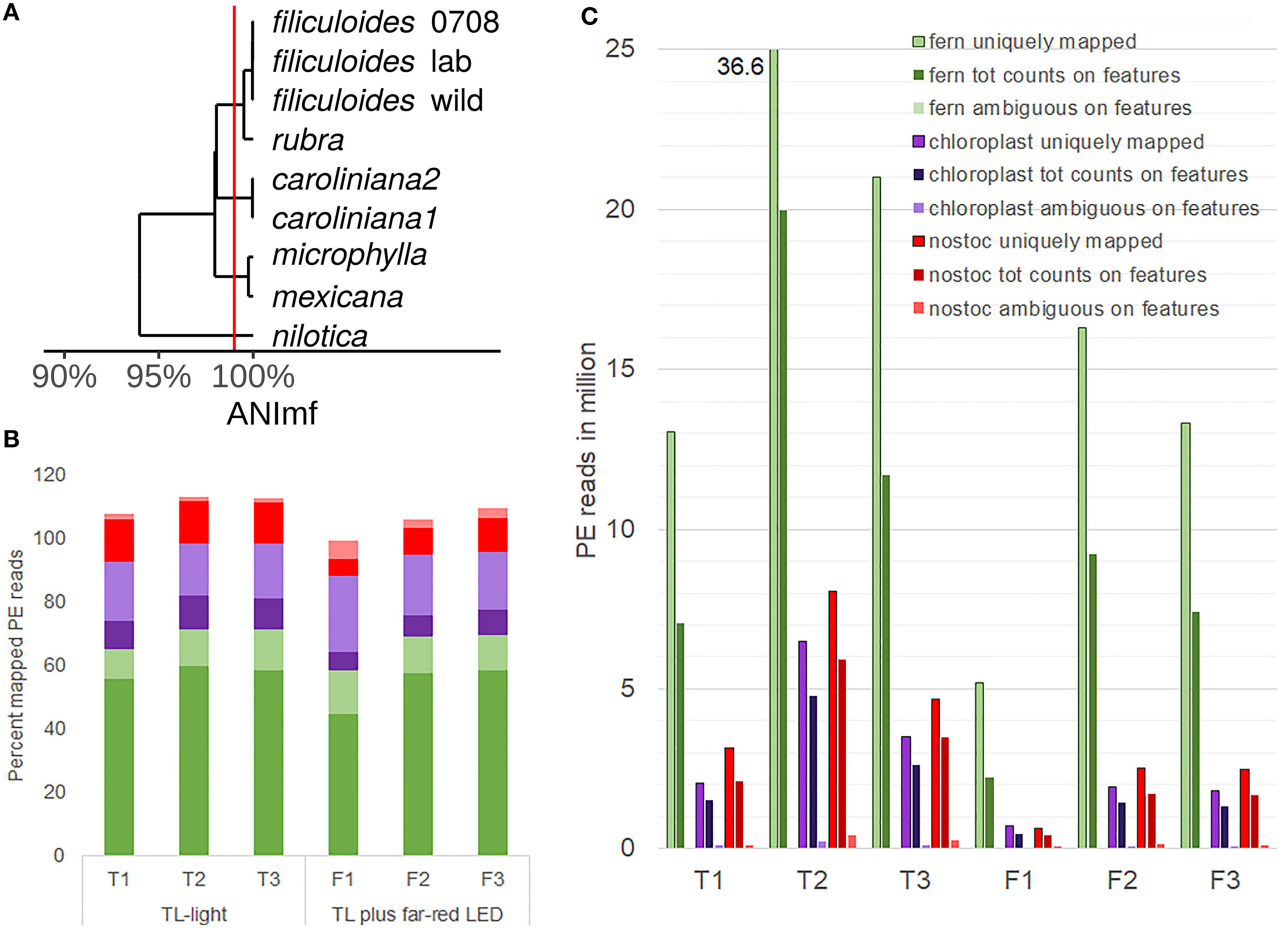
Profiling eukaryotic and prokaryotic RNA simultaneously in *A. filiculoides* [dual RNA**-**sequencing (RNA-seq)]. **(A)**, Similarity of the *N. azollae* 0708 genome and metagenome assembled genomes (MAGs) from *N. azollae* strains in *Azolla* species. Average nucleotide identity (ANI) was calculated with the dRep implementation of nucmer using the ANImf preset (Kurtz et al., 2004; Olm et al., 2017). Pairwise average ANI is shown in an UPGMA dendrogram (Wickham, 2011; de Vries and Ripley, 2016). Aligned MAGS covered over 90% of the reference *N. azollae* 0708. **(B)** Proportion (percent) of uniquely aligned paired-end (PE) reads (dark color) and PE reads aligned at multiple loci (light color) on the genomes of fern (green), fern chloroplast (purple), and *N. azollae 0708* (red). PE reads were from the samples of sporophytes grown for 1 week on TL light (replicates T1, T2, and T3) or TL light with FR (replicates F1, F2, and F3). STAR-aligner was used in PE mode. **(C)** Yields of PE uniquely mapped were compared with PE read counts on features and PE reads that STAR was aligned ambiguously to several features. Features were specified by current general features files of each of the fern (green), chloroplast (purple), and *N. azollae* 0708 (red) genomes.

The *N. azollae* Galgenwaard genomes were assembled both from *A. filiculoides* kept in the laboratory (*A. filiculoides* lab; Li et al., 2018), taken from the original sampling location (*A. filiculoides* wild; Dijkhuizen et al., 2018) and other *Azolla* species (Dijkhuizen et al., 2018). Assemblies were obtained from the short reads of the entire symbiosis metagenome (MAGs); they were fragmented but highly complete: over 95% complete based on single copy marker genes. Moreover, all contigs of *N. azollae* from the Utrecht *A. filiculoides* aligned to the *N. azollae* 0708 reference genome from *A. filiculoides* Stockholm (Ran et al., 2010) and were well over 99.5% identical to each other (Figure 4A). This degree of nucleotide identity was well above the maximum mismatch of the default setting of STAR-aligner default setting, which meant that reads could be aligned to the reference taking the NCBI-defined features to derive read counts for each feature: NCBI-defined features we argue are superior to the automated annotation by Prokka (Seemann, 2014).

The PE reads obtained from sequencing libraries made from rRNA-depleted RNA were thus aligned to the Galgenwaard accession nuclear and chloroplast genomes, and the *N. azollae* 0708 genome, and the read counts derived from their GFF annotation files. Alignments revealed a high proportion of uniquely mapped reads to the fern nuclear and *N. azollae* genomes suggesting an efficient removal of rRNA (Figure 4B). For the chloroplast, a larger proportion of PE reads mapping to multiple loci in the chloroplast was mostly due to the repeated genes with a minor contribution of reads from the chloroplast 23S rRNA sequence making up no more than 0.35% of the reads in the six samples. Read counts for the few chloroplast genes were generally high (84 without rRNA and tRNA genes). Read counts in genome features further revealed that the sense alignments dominated and thus that the libraries were stranded (Figure 4C).

Dispersion of sense read count per gene was low, which permitted the identification of differentially accumulating transcripts [defined as DEseq2 *P*adj < 0.1; Love et al., 2014] comparing the ferns on TL and TL with FR: 318 from the fern nucleus, 67 from *N. azollae*, and 5 from the fern chloroplast (Supplementary Table 4). We next wondered whether the changes in transcript abundance reflect the adaptations to light quality and the transition to reproductive development.

### FR Causes Small Transcriptional Changes That May Reflect Small Light-Harvesting Adaptations in Chloroplasts and *N. azollae*, but Large Changes in Transporters of *N. azollae*

After 1-week exposure to FR, decreased chloroplast transcripts included *psbD* (18651 BM, −0.72 log 2-fold change (log2FC), 0.074 *P*adj), previously reported as responsive to light quality in plant lineages (Shimmura et al., 2017) and suggesting that the obtained read counts made some sense. In contrast, increased chloroplast transcripts encoded *ycf2* [141 base mean (BM) related to reads per million (this is low because ycf2 is in the repeat region), 2.9 log2FC, 0.001 *P*adj], energizing the transport of proteins into the chloroplast, and the RNA polymerase subunit *rpoA* (1,284 BM, 1.3 log2FC, 0.000 *P*adj) (Supplementary Table 4, *A. filiculoides* chloroplast).

Consistent with the high levels of N_2_ fixation measured in Azolla (Brouwer et al., 2017), the highest *N. azollae* read counts were in the transcripts of the *nif* operon [the highest: AAZO_RS06505 *nifH* averaging 45,084 reads per million reads aligned to the N. *azolla*e genome (rpm)], genes encoding photosystem I (AAZO_RS02645 *psbA* averaging 47,415 rpm) and II proteins (AAZO_RS06500 *psbB* averaging 29,767 rpm), and ATP synthase subunits (the highest: AAZO_RS15835 *AtpA* averaging 13,024 rpm). In addition, read counts for the global nitrogen regulator *ntcA* (AAZO_RS05170 averaging 1,329 rpm) and metabolic enzymes supporting high N_2_ fixation were high (Supplementary Table 5). Read counts from the *N. azollae* transcripts reflected the known very high N_2_-fixation capacity of the symbiont; we thus analyzed the data for differential expression with a measure of confidence.

In *N. azollae*, FR led to barely significant accumulation of a *PsbA* transcript of low abundance (817 BM; 1.36 log2FC with 0.099 *P*adj; Supplementary Table 4, *N. azollae*). In contrast, FR led to a significant reduction of transcripts from photosystem I reaction center subunit XII (149 BM; −1.22 log2FC, 0.047 *P*adj), geranylgeranyl hydrogenase *ChlP* (909 BM; −1.10 log2FC; 0.037 *P*adj), and phycobilisome rod-core linker polypeptide *CpcG2* (1,588 BM; −1.19 log2FC; 0.002 *P*adj; Supplementary Table 4, *N. azollae*). These changes in light-harvesting-related transcripts were small, however, compared to changes in transcripts encoding the hypothetical proteins of unknown function, transposases, and highly expressed transporters (*TrKA*: 2,355 BM, 3.41 log2FC, 0.000 *P*adj; *MFS*: 2,361 BM, 2.66 log2FC, 0.003 *P*adj; and ABC transporter permease: 3,996 BM, 2.53 log2FC, 0.000 Padj). We conclude that a 1-week induction of reproductive structures with FR may have caused a few and small transcriptional changes reflecting light-harvesting adaptations in *N. azollae*. A larger differential accumulation of transporter transcripts may reflect more important changes in metabolite trafficking and communication with the host fern.

### FR Alters Transcripts From MIKC^C^ and R2R3MYB TF Related to Those of the IF of Seed Plants

As in the case of *N. azollae* symbiont, the largest changes of fern-transcript accumulation under FR were noticed in genes with an unknown function, then in secondary- and lipid-metabolism (Supplementary Table 4, *A. filiculoides* nucleus). We noted a large induction of the SWEET-like transporter (Azfi_s0221.g058704: 22 BM, 7.5 log2FC, 0.000 *P*adj), of the transcripts related to cysteine-rich peptide signaling (Azfi_s0046.g030195: 42 BM, 7.1 log2FC, 0.074 *P*adj; Azfi_s0745.g084813: 99 BM, 4.0 log2FC, 0.000 *P*adj) and of the transcripts from jasmonate metabolism. To examine the link between IF in seed plants and IS ferns, however, TFs were analyzed in more depth. The transcripts of MIKC^C^ and of R2R3MYB TF were strikingly induced in sporophytes under FR (Table 2).

**Table 2:** Transcription factors (TFs) with largest changes in transcript abundance in sporophytes in tube light (TL) with vs. without far-red (FR).

***A. filiculoides* locus**	**baseMean**	**Log2 fold change**	**DESeq2 *p*adj**	**Mercator 4.0 annotation**	**Closest Arabidopsis Homolog**
**Azfi_s0028.g024032**	**9**	**7.22**	**0.001**	**15.5.14 MADS/AGL**	**AGL20/SOC1, MIKC** ^**C**^
Azfi_s0113.g045874	155	6.41	0.002	15.5.2 R2R3MYB	AtMYB 32 (AT4G34990.1), R2R3MYB VIII-E^*^
Azfi_s0083.g038807	59	5.75	0	15.5.32 Basic Helix-Loop-Helix	EDA33, IND1, INDEHISCENT (AT4G00120.1)
Azfi_s0003.g007560	8	5.27	0.011	15.5.32 Basic Helix-Loop-Helix	FIT1 (regulates iron transport)
Azfi_s0003.g007559	34	3.49	0	15.5.32 Basic Helix-Loop-Helix	FIT1 (regulates iron transport)
Azfi_s0096.g043715	31	3.13	0.001	15.5.7 DREB subfamily A-2 of ERF/AP2	AT5G18450.1
**Azfi_s0003.g007710**	1,778	2.82	0	**15.5.14 MADS/AGL**	**AGL6, MIKC** ^**C**^
Azfi_s0015.g013719	111	2.57	0	15.5.2 G2-like, GARP	HHO2, NIGT1.2
Azfi_s0112.g045798	66	2.2	0	15.5.51.1 NF-Y component NF-YA	AT5G12840.4
Azfi_s0014.g013539	105	2.07	0.005	**15.5.2 R2R3MYB**	LOF2, R2R3MYB V^*^
Azfi_s0015.g014012	165	1.99	0.026	15.5.17 NAC domain	NAC025
**Azfi_s0004.g008455**	**103**	**1.93**	**0.003**	**15.5.2 R2R3MYB**	miRNA319 controlled GAMYB 33, R2R3MYB VII^*^
Azfi_s0132.g049213	63	−2.35	0.004	15.5.32 Basic Helix-Loop-Helix	No hits
Azfi_s0182.g056462	10	−4.46	0.049	15.5.3 HD-ZIP I/II	HB16

*TF with homology to TF related to flowering are bold type*.

* *R2R3 MYB classification according to Jiang and Rao (2020)*.

The most induced TF transcript was annotated as SOC1-like MIKC^C^ (Azfi_s0028.g024032: 9 BM, 7.2 log2FC, 0.001 *P*adj); its predicted gene model, however, lacked the K-domain. This problem was due to the fact that the TF encoded by Azfi_s0028.g024032 was not expressed in vegetative sporophytes used for the original annotation of the *A. filiculoides* genome (Brouwer et al., 2017; Li et al., 2018). Manual re-annotation of this TF using the reads obtained in this study allowed to place it precisely in the phylogenetic trees of MIKC^C^ TFs sampled over taxa representing the different lineages of land plant evolution (Supplementary Figure 4). In fact, both induced Azolla MIKC^C^ from Table 2 were placed in the reproducibly well-supported clade (95.5% bootstrap confidence) of fern sequences radiating separately from the angiosperm and gymnosperm MIKC^C^ clade that gave rise to AtSOC1, FLC, and the A-, C-, D-, and E-function floral homeotic genes (Supplementary Figure 4). Induction of the *A. filiculoides* SOC1-paralog was confirmed by qRT-PCR (Supplementary Figure 2G). In Arabidopsis, AtSOC1 is known to control the steady state of miR319 that inhibits TCP TF functions necessary for the transition to an inflorescence meristem at the shoot apex (Lucero et al., 2017); the link may also operate in Azolla as a TCP TF (Azfi_s0168.g054602: 661 BM, 1.11 log2FC; 0.086 *P*adj) was induced in ferns on TL with FR (Supplementary Table 4, *A. filiculoides* nucleus).

The transcripts of R2R3MYB TFs could be assigned to families using the data from Jiang and Rao (2020) (Table 2). The highly induced class VIII-E TF (Azfi_0113.g045874: 155 BM, 6.4 log2FC, 0.002 *P*adj) likely controls the changes in secondary metabolism together with the similarly changed bHLH TF (Güngör et al., 2020), and therefore may not be directly linked to IS. In contrast, induced R2R3MYB from the classes V (Azfi_s0014.g013539: 105 BM, 2.1 log2FC, 0.005 *P*adj) and VII (Azfi_s0004.g008455: 104 BM, 1.9 log2FC, 0.003 *P*adj) are known to affect seed plant IF, with the GA pathway-associated class VII GAMYB in seed plants known to be regulated by miR319.

Prominence of TFs in Table 2 known for their role in IF-development in seed plants begged the question as to whether any IF pathways with conserved miRNA were conserved in the common ancestor of seed plants and ferns. The question required, however, to first characterize miRNA loci in the *A. filiculoides* symbiosis.

### Small RNA Profiles Are Characteristic for Fern Nucleus, Chloroplast or *N. azollae*

**Figure 5:**
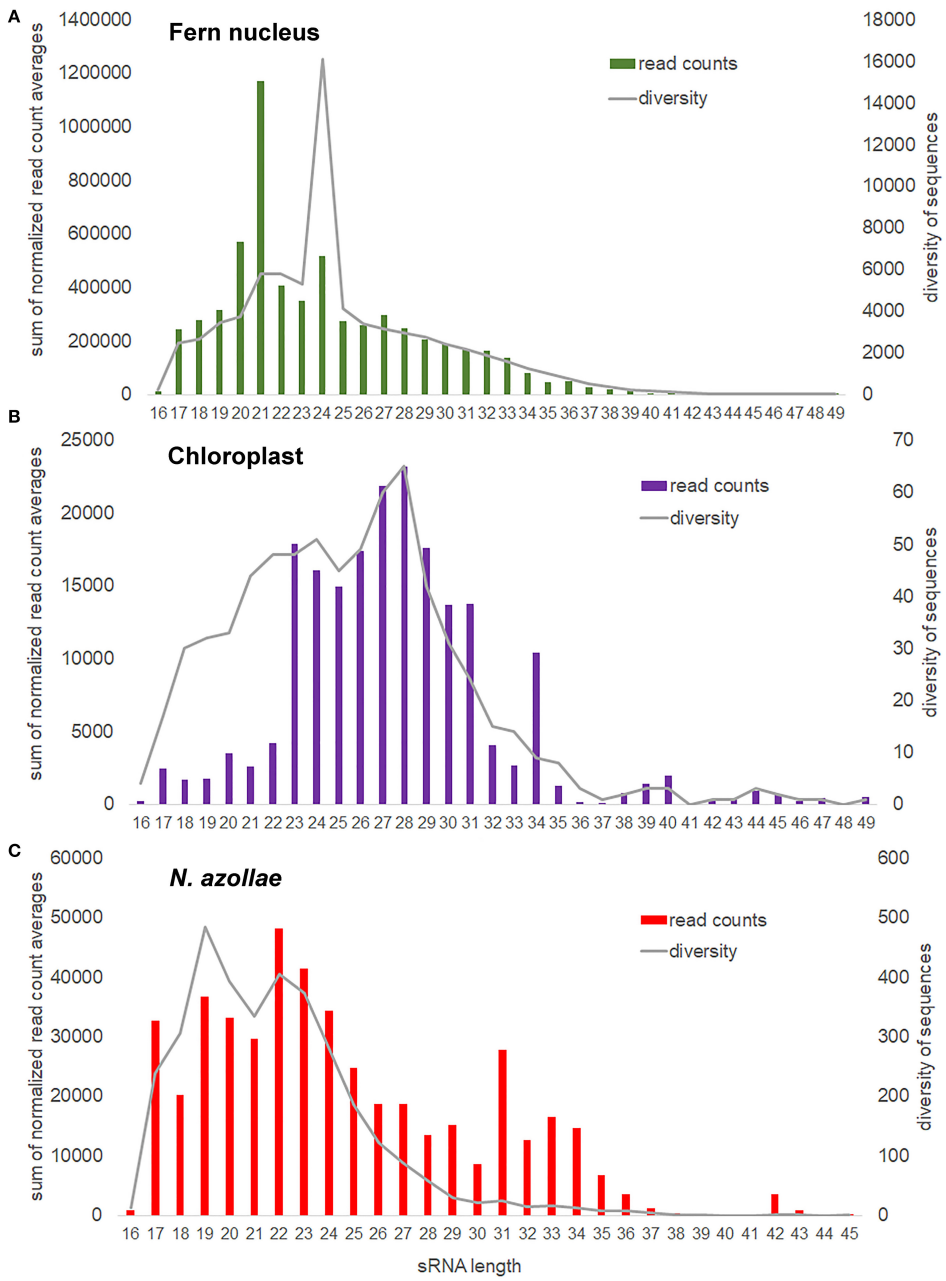
Small RNA (sRNA) size distribution and sequence diversity for each size from the genomes in the *A. filiculoides* symbiosis. sRNA reads were mapped with zero mismatches, no overhang, to the concatenated genomes; bam2fasta was then used to extract sRNA sequences aligned per genome, the obtained fasta files were then collapsed to compute read counts for each sRNA, the obtained lists were then joined to a read count matrix imposing at least one read per sRNA in each of the three replicate sample types: f (FR), t (TL), and fn (FR and nitrogen in the medium). sRNA counts were then normalized to the total read counts per sample for that genome. sRNA read counts were further averaged over all samples considered, then the averages with the same size summed up, which yielded the sum of normalized read count averages and reflects how many sRNA are found for each size (read counts). For each sRNA size, we also calculated the mean number of different sRNA sequences found (diversity). **(A)** Fern nucleus. **(B)** Chloroplast, included the samples from sporophytes without cyanobacteria in the analysis. **(C)** *N. azollae*, read count files were joined imposing at least 10 reads per sRNA in every sample.

Short RNA reads were obtained from sporophytes after 1 week growth under TL without and with FR from the same cultures as those used for dual RNA-seq. In addition, reads were obtained from the sporophytes grown under TL with FR on medium with 2 mM NH_4_NO_3_, and from the sporophytes grown without *N. azollae* on medium with 2 mM NH_4_NO_3_. All samples were collected 2 h into the 16 h light period. The small-RNA sequencing library preparation chemistry did not distinguish prokaryotic sRNA from eukaryotic sRNA and therefore was inherently a dual profiling method. The obtained reads were aligned to concatenated genomes, which yielded sRNA characteristic profiles for the fern nucleus, chloroplast, and *N. azollae* (Figure 5; Supplementary Figure 5).

The most abundant sRNAs encoded in the fern nucleus were 21, 20, and 24 nt long, but the most sequence diverse sRNAs were 24 nt long (Figure 5A). This was consistent with an abundance of 21-nt miRNA discretely cut into one or a very few 21-nt mRNAs from a single hairpin precursor (Singh et al., 2018). The higher sequence diversity of the somewhat less abundant 24-nt small interfering RNAs (siRNAs) was consistent with the multiple cleavage sites on the longer double-stranded RNA generated by RNA-dependent polymerase of the siRNA precursors that lead to several different 24-nt siRNAs per locus transcribed (Singh et al., 2018). The most abundant chloroplast sRNAs of 28 nt were also the most sequence diverse ones, yet, the chloroplast sRNAs of 34 nt were surprisingly numerous and comparatively little diverse in sequences (Figure 5B). The sRNAs encoded in *N. azollae* were generally shorter than those of the chloroplast, they had a conspicuous abundance of 42-nt sRNAs, consistent in length with CRISPR RNA but inconsistent in diversity of sequence for a function in immunity (Figure 5C).

### Conserved miRNAs in *A. filiculoides* Include miR172 and 396, but Only miR319 and miR159 Were Decreased in Response to FR, While miR529 and miR535 Were Increased

**Figure 6:**
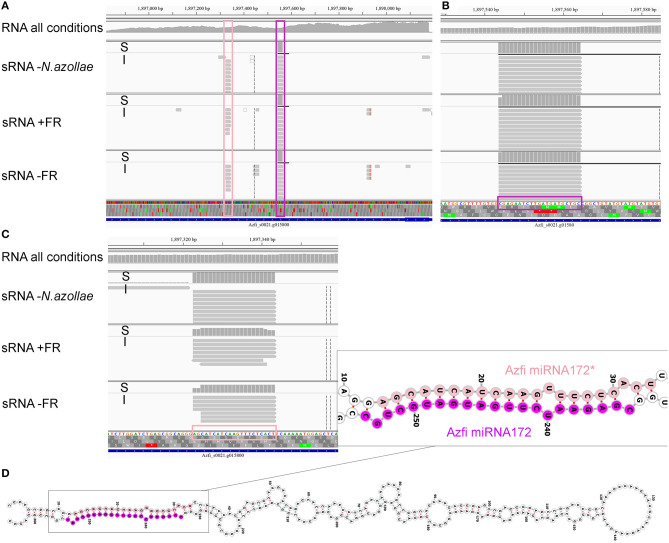
The microRNA (miRNA) 172a locus in *A. filiculoides*. Alignments at the Azfi_miRNA172a locus are visualized using the Integrative Genomics Viewer (Thorvaldsdóttir et al., 2013); S, read summary; I, individual reads. **(A)** Reads from pre-miRNA at the miR172a locus. RNA-seq all conditions, all reads pooled from the dual RNA-seq experiment in Brouwer et al. (2017). **(B)** Reads of the miR172 in sRNA libraries including those of ferns without *N. azollae* (sRNA-seq—*N. azollae*), ferns grown with and without FR (sRNA-seq+FR, sRNA-seq TL-FR). **(C)** Reads of the miR172*. **(D)** Azfi_miRNA172a hairpin folded using Vienna RNAfold (Gruber et al., 2008; predicts −104.07 kcal mol^−1^ at 22°C upon folding); miRNA (purple) and miRNA* (pink) stem arms are shown with 3'overhangs.

Fern genome-encoded sRNA reads of 20–25 nt length were used for the miRNA predictions allowing a hairpin of maximally 500 bp. The predicted loci were then verified against the miRNA criteria (Axtell and Meyers, 2018) as, for example, in the case of the miR172a locus Azfi_s0021g15800 (Figure 6): the locus was expressed as a pre-miRNA (Figure 6A, RNA-seq all conditions) and both the miRNA and miRNA^*^ are found in replicate samples and cut precisely with two base overhangs (Figures 6B,C, sRNA-seq). The miR172 reads were derived from the fern as they were also found in sporophytes without *N. azollae* (Figure 6, RNA-seq *N. azollae*). The locus encoded a stable long hairpin, which, when folded, released −65.76 kcal mol^−1^ at 37°C. The miR172 targets in seed plants include the AP2/TOE TFs, and the alignment of the target sites to all annotated AP2/TOE transcripts from *A. filiculoides* revealed the target sites for AzfimiR172 (Supplementary Figure 6). The miR156a locus was similarly verified (Supplementary Figure 7).

**Figure 7:**
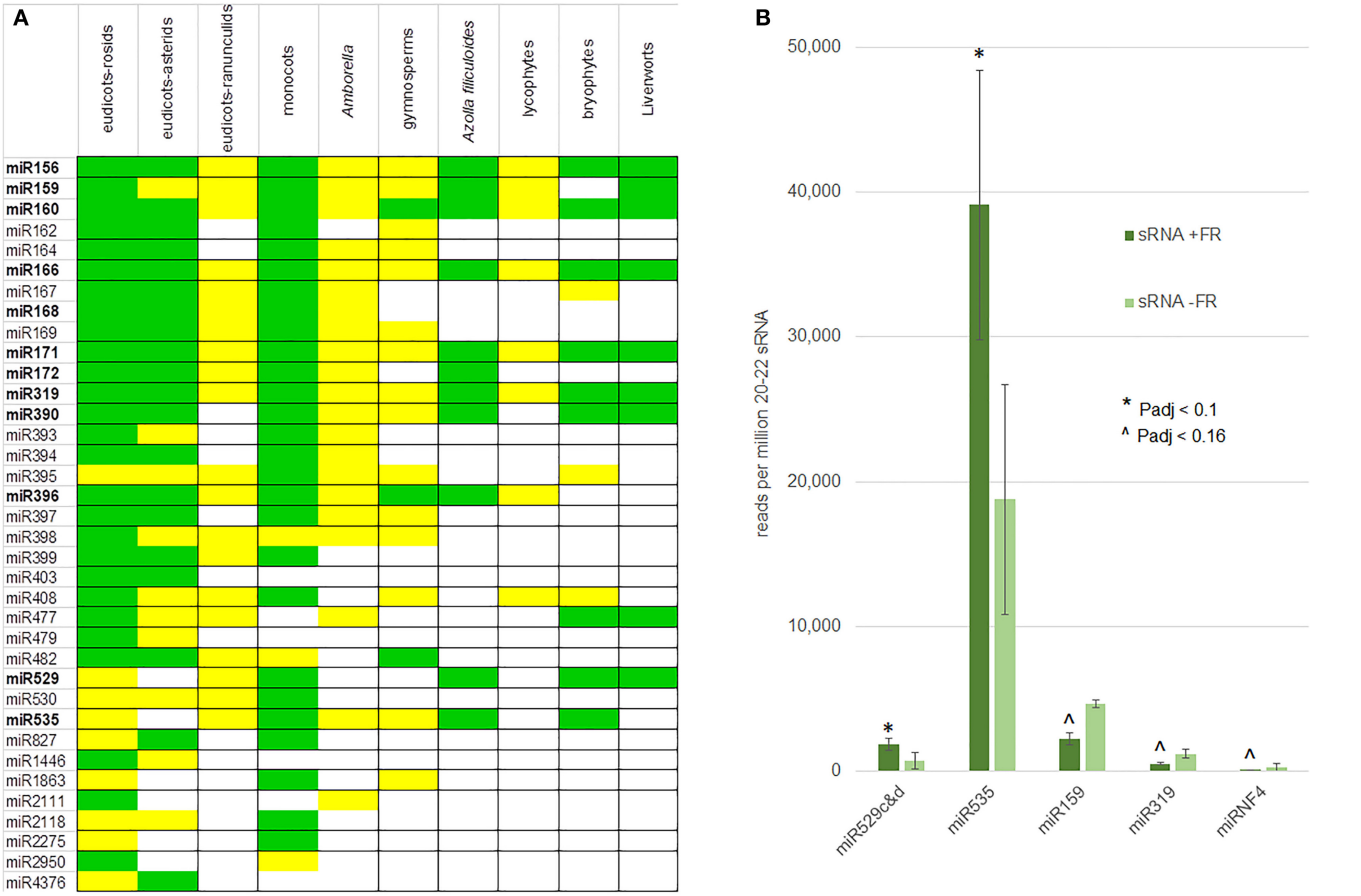
Conserved and FR-responsive miRNA in the fern *A. filiculoides*. **(A)** Conserved miRNA in the *A. filiculoides* fern compared to other land plant lineages. miRNAs from the fern were predicted computationally by miRDEEP-P2 (Kuang et al., 2019) using sRNA-seq reads and then curated manually, the remainder of the table was from Axtell and Meyers (2018). Green, high confidence miRNA; yellow, low confidence miRNA. **(B)** miRNA with altered steady-state in response to FR light. The average read counts per million 20–22 nt reads in the samples of sporophytes after 1 week on TL with FR (sRNA + FR) or without (sRNA − FR) are shown; raw read counts were submitted to DESeq2 for statistical analyses yielding *P*-adjusted (P-adj) values: * indicates *P*adj < 0.1; ∧ indicates *P*adj < 0.16.

Mature miRNAs were compared in miRbase (Kozomara et al., 2019), which identified 11 of the conserved miRNA families of 21 nt in the *A. filiculoides* genome and thus in the fern lineage (Figure 7A). The list included miR396, as well as miR172, not yet confirmed in other seed-free plant lineages. The miRNA predictions further uncovered a number of novel miRNAs (miRNF).

Differential expression analysis of 20–22 nt reads exhibited more dispersion than the dual RNA-seq of long RNA, yet, it reliably identified sRNA with low *P*adj values when comparing the ferns with and without FR under TL (Supplementary Figures 5D,E). The reads from miR529c&d and miR535 increased (*P*adj < 0.1) while those of miR159, miR319, and miRNF4 decreased (*P*adj < 0.16) in sporophytes on TL with FR compared to TL light only (Figure 7B). We conclude that the miR156/miR172 known to reciprocally control flowering do not accumulate differentially when profiling whole ferns undergoing transition to IS under FR is analyzed.

### miR319/GAMYB Is Responsive in FR-Induced Sporophytes

miRNA targets were predicted by combining psRNATarget (Dai et al., 2018) and TargetFinder (Srivastava et al., 2014) and then annotated with Mercator (Lohse et al., 2014; Schwacke et al., 2019). The predictions were consistent with analyses in other land plant lineages (Supplementary Table 6): miR172 targeted AP2/TOE-like TFs and miR156, miR529, and miR319 SPL-like proteins.

Transcripts of neither SPL nor AP2/TOE-like targets, however, were significantly (*P*adj < 0.1) altered in sporophytes comparing TL with and without FR (Supplementary Table 6, F vs. T *P*adj). The Azfi vs1 assembly is far from the chromosome scale, and its annotation is based on a very few experimental datasets, we may therefore be missing SPL or AP2 targets. Nevertheless, consistent with an increased miR529c&d, the SPL from the Azfi_s0173.g055767 transcript appeared to be reduced but with no significance (*P*adj of 0.356). In contrast, transcripts of two GAMYB (Azfi_s0004.g008455 and Azfi_s0021.g015882) increased up to four-fold in sporophytes with decreased miR319 on TL with FR. The location of miR319 binding for GAMYB encoded in Azfi_s0004.g008455 is shown (Supplementary Figure 8).

**Figure 8:**
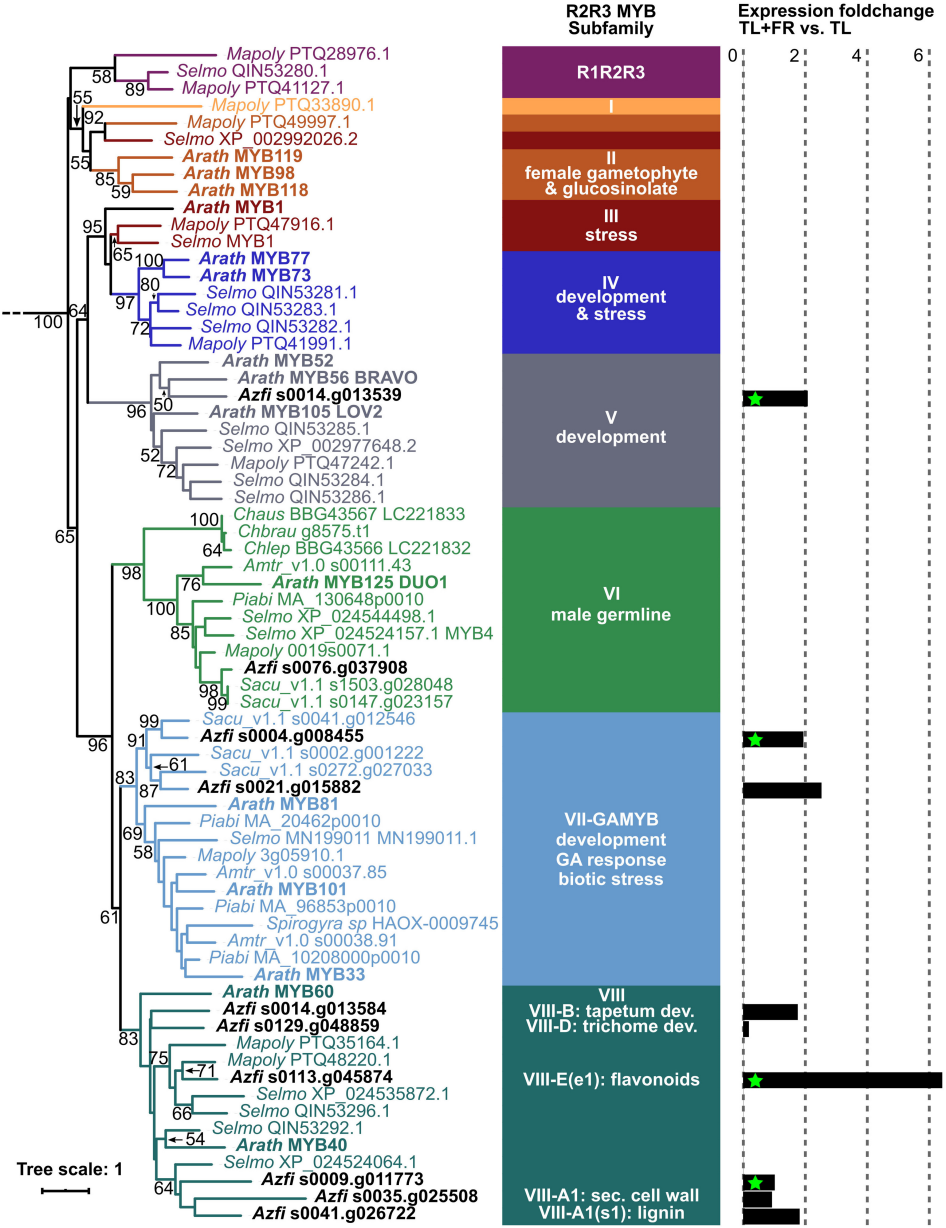
R2R3MYB phylogenetic analyses and expression fold change in sporophytes with vs. without FR. Sequences were extracted from the genome browsers of each species, aligned with MAFFT E-INS-I (Katoh et al., 2019), and then trimmed with trimAL (gap threshold 40%; Capella-Gutiérrez et al., 2009). Phylogenetic inferences were computed with IQtree with the LG+F+R10 model as determined by the Iqtree internal model fitter (Nguyen et al., 2015); non-parametric bootstrap values are shown. *Chara autralis* (Chaus), *Chara braunii* (Chbrau), *Chara leptospora* (Chlep), *Marchantia polymorpha* (Mapoly), *Selaginella moellendorfii* (Selmo), *A. filiculoides* (Azfi), *Salvinia cuculata* (Sacu), *Picea abies* (Piabi), *Amborella trichopoda* (Amtr), and *Arabidopsis thaliana* (Arath). Sequence names are color coded after the R2R3 MYB subfamilies defined in Jiang and Rao (2020). Log two-fold change (fold change) in response to FR was calculated by DESeq2, green stars mark significant changes with *P*adj < 0.1.

Phylogenetic analyses revealed that the GAMYB targeted by miR319 were on the same leaf as GAMYB33 from Arabidopsis and they were placed next to each other probably because they arose from gene duplication (Figure 8). This is consistent with the whole genome duplication observed at the base of Azolla fern evolution (Li et al., 2018). We conclude, therefore, that the miR319/GAMYB regulon is responsive in sporophytes undergoing FR-induced transition to IS.

## Discussion

The obtained results indicated that FR and canopy density are required for the elongation and then the induction of sporocarps in *A. filiculoides;* the sporocarps thus obtained could be used for crosses. The results were valid for *A. filiculoides* but not for a different species collected in the Anzali lagoon, phylogenetically close to the species from Southern Brazil and Uruguay. Fern and seed plants share a common ancestor, which may predict similar mechanisms in the responses to FR, including the elongation response and the transition to sexual reproduction. Dual RNA profiling revealed a differential transcript accumulation in the symbiosis upon exposure to FR, most notably of transporters in the *N. azollae*, and of TF known from the flowering transition in seed plants. sRNA profiling provided a first insight into the diversity of sRNA of the symbiosis including conserved miRNA in the fern and other land plant lineages. Thus, first, we discuss the possible mechanisms involved in the sensing and signal transduction of FR in ferns compared to other plant lineages. Second, we discuss the regulons that appeared to be conserved in ferns and seed plants undergoing the transition to sexual reproduction. Finally, we discuss the method of dual RNA-seq as a more generally required but an insufficient tool.

### Red-Dominated Light Suppresses the Formation of Dissemination Stages in Both Gametophyte- and Sporophyte-Dominated Lineages of Plants and Represents a Convergent Ecological Strategy

Far-red responses in Azolla look like a shade-avoidance syndrome but the signal transduction pathways that mediate them in ferns remain largely uncharacterized (Inoue et al., 2019). Pathways causing shade response components are known to use alternative phytochromes and interacting factors (Possart and Hiltbrunner, 2013; Xie et al., 2020).

Phytochromes in ferns radiated separately from those of seed plants (Li et al., 2015), hence, their function cannot be predicted using orthology with seed plants. Nevertheless, fern phytochromes have a similar structure and, as in the case of PHYB in Arabidopsis, may sense temperature as well as red/FR light by thermal reversion from the *P*_fr_ into *P*_r_ state (Legris et al., 2017; Klose et al., 2020): initiation of fern, liverwort, and bryophyte sporangia is reported as temperature- and photoperiod-dependent (Labouriau, 1958; Benson-Evans, 1961; Nishihama et al., 2015). Moreover, thermal reversion of phytochromes from some cyanobacteria was described *in vitro* as early as 1997 (Yeh, 1997). Therefore, if FR induces IS in *A. filiculoides*, then differing temperature regimes combined with light quality changes may yet induce IS in the Anzali ferns. Anzali ferns were not *A. filiculoides* but related to the species from Uruguay and Southern Brazil, which did not cluster with the bulk of the *A. caroliniana* species (Figure 2), and did not have the single-cell papillae described for *A. caroliniana* (Pereira et al., 2001; Supplementary Figure 3). More work is needed to verify whether these South American strains represent a new species of *Azolla*. The phylogenetic position of the Anzali accession analyzed here was consistent with the observations of several distinct-looking Azolla in the Anzali lagoon (Farahpour-Haghani et al., 2017), and suggested that not only *A. filiculoides* may have been released as nitrogen biofertilizer.

Unlike *A. filiculoides*, Arabidopsis will make the transition to IF in TL generally used for indoor cultivation. Most studies on the transition to IF, therefore, were done with red-light gated Arabidopsis plants, in conditions leading to artifactual peaking of CONSTANS protein accumulation and florigen (FT) expression (Song et al., 2018). Nevertheless, Arabidopsis as well as many other seed plants flower early when the ratios of red to FR approach 1, the ratio encountered in day light, compared to TL. Far-red-dependent flowering is mediated by PHYB and D, eventually leading to *SOC1* expression in the meristem (Halliday et al., 1994; Aukerman et al., 1997; Lazaro et al., 2015). *ft* mutant Arabidopsis flowered earlier under incandescent light (Martinez-Zapater and Somerville, 1990) or in FR compared to in TL (Schluepmann et al., unpublished) suggesting that FT is not involved. Signaling was also reported *via* PHYB-regulated PIF4 protein complexes that directly bind the promoters of miR156/172 with an interference from PHYA-regulated TF (Sánchez-Retuerta et al., 2018; Sun et al., 2018; Xie et al., 2020). In the present study, the highly induced MIKC^C^ turned out to be a paralog of AtSOC1, and the miR156/172 steady states were unaltered by FR-induced transition to IS in *A. filiculoides* (Figure 7B; Supplementary Table 6) suggesting that signal transduction leading to IS/IF control in fern and seed plant sporophytes are not conserved.

Far red light induced sexual reproduction in the gametophytes of the liverwort *Marchantia polymorpha* (Chiyoda et al., 2008; Kubota et al., 2014; Yamaoka et al., 2018; Tsuzuki et al., 2019). The mechanism in these liverworts involved the MpmiR529c/MpSPL module, which was necessary for the development of reproductive branches. MpPHY and MpPIF were required to mediate the response (Inoue et al., 2019). Our analyses using *A. filiculoides* reveal the induction of AzfimiR529 under FR but no significant change in SPL targets. Additionally, the specific MpmiR529c sequence involved in *M. polymorpha* was not detected in our ferns. The pathways thus differ in the two seed-free plants, unsurprisingly given that one operates in sporophytes and the other in gametophytes (Hisanaga et al., 2019).

Given that gametophyte- and sporophyte-dominated lineages require FR to initiate the formation of dissemination stages, we conclude that the repression of IS under dominating red light in Azolla likely reflects a convergent ecological strategy: in open surfaces, where red-light is abundant, there is a sufficient space to prolong vegetative development and no need for dispersal. In addition to FR, the density of the *A. filiculoides* canopy had an additional impact on the number of sporocarps observed per gram plant (Figure 2F); this may stem from alternative cues such as volatile organic compounds released by neighbors (Vicherová et al., 2020). Taken together, the results presented in this work signify that, for the first time, IS of the Azolla fern symbiosis is amenable to experimental enquiry.

### Phylogenetic Position of MIKC^C^ TF Responsive to IS Suggests That the Control of Flowering in Seed Plants Originates From the Diploid to Haploid Transition in the Common Ancestor of Seed Plants and Ferns

The GAMYB TF clade arose before the land plants evolved, and before GA signaling (Aya et al., 2011; Bowman et al., 2017; Jiang and Rao, 2020; Figure 8). The Azolla GAMYB induced under FR, therefore, likely mediate cues perceived by the sporophyte but acting upon functions originating from an ancestral gametophyte regulon. Consistently, GAMYB from the moss *Physcomytrella patens* is required for the formation of antheridiophores by the gametophyte (Aya et al., 2011). The lycophyte GAMYB regulon did not play a role in the induction of sporogenic structures of the sporophyte.

AtGAMYB is known to be regulated by miR159 in Arabidopsis and by the related and more ancestral miR319 in seed-free plant lineages (Achard et al., 2004; Palatnik et al., 2007). The AtGAMYB/miR159 is part of the GA pathway promoting flowering in Arabidopsis under short days (Millar et al., 2019). Specifically, AtMYB33 was shown to directly act on the promoters of both miR156 and AtSPL9 (Guo et al., 2017). In Azolla, FR repressed miR159 and miR319, and increased AzfiGAMYB but this is difficult to interpret given the compressed life cycle of the gametophyte inside the sporocarps. Extending the analysis of the GAMYB regulon to homosporous ferns with free gametophytic stages will reveal whether this regulon is important for the diploid to haploid transition, or rather functions solely in the gametophyte development as predicted from lycophyte studies.

The control over sexual reproduction was switched from the gametophyte, in gametophyte-dominated seed-free plants, to the diploid sporophyte in seed plants. The switch led to the evolution of regulons with MIKC^C^ TF commanding floral meristems and flower architecture. The MIKC^C^ clade of TF that specify both IF and floral organs in seed plant sporophytes (such as AtSOC1 and AtFLC, and the A,C, D, and E homeotic functions) is peculiar in that the TF radiated in each megaphyll plant lineage separately (Leebens-Mack et al., 2019; Supplementary Figure 4). MIKC^C^ of angiosperms were reported to work as hetero-tetramers and may have evolved from an ancient tandem gene duplication through subsequent polyploidy events (Zhao et al., 2017). Sepals are organs formed only in angiosperms. Consistently, the TF clade with A functions specifying sepals contains solely angiosperm sequences. MIKC^C^ specifying the organs that contain the reproductive structures in angiosperms (C, D, and E homeotic genes) had direct homologs in gymnosperms but not in ferns. Instead, ferns radiated a sister clade to the clade containing AtSOC1 and A, C, D, and E MIKC^C^. This clade contained the Azolla MIKC^C^ responsive to FR (SOC1-paralogs) and the closely related CMADS1 from *Ceratopteris richardii*. *In situ* hybridizations and northern blot analyses revealed that *CMADS1* transcripts accumulate at high levels in the sporangia initials (Hasebe et al., 1998). At the base of the radiating clade of modern MIKC^C^ that specify flower organs of the sporophytes controlling the gametophyte development within, is the LAMB1 protein expressed specifically in the sporogenic structures of lycophytes (Svensson et al., 2000; Leebens-Mack et al., 2019; and Supplementary Figure 4). Combined phylogenetic and RNA-seq analyses, therefore, suggest that the MIKC^C^ regulons controlling flowering and flower architecture likely originate from the diploid to haploid phase transition of the common ancestor to ferns and seed plants, not from regulons controlling sexual reproduction *per se*.

### Dual RNA-seq to Study Coordinate Development in Assemblages of Prokaryotic and Eukaryotic Organisms

The dual RNA-seq method employed here could be applied more generally to study bacteria/plant assemblages common in the seed-free plant lineages, and representing an important base of the “tangled tree” of plant lineage evolution (Quammen, 2018). For Azolla where coevolution of *N. azollae* and fern host genomes demonstrated that fitness selection occurs at the level of the metagenome (Li et al., 2018), it delivered foundational data and opens the way to studying the coordinate development of host and obligate symbiont. Efficient removal of the rRNA was a key to the success of sequencing RNA. Transcripts from the mitochondrial genome of fern are likely in the data but we have yet to assemble the *A. filiculoides* mitochondrial genome to confirm this.

The RNA profiling did not reveal whether the *N. azollae* trigger fern IS as the roles of the FR-responsive transcripts in *N. azollae*, particularly those encoding the transporters, have yet to be studied. An approach could be to study these in free living filamentous cyanobacteria amenable to genetic manipulation. The manipulation of the symbiont would, however, be preferred. Tri-parental mating protocols may be effective on *N. azollae in situ* if combined with high-frequency RNA-guided integration with Cas12k enzyme complexes (Strecker et al., 2019). *N. azollae* Cas genes were found to be mostly pseudogenes, and CRISPR arrays appeared missing (Cai et al., 2013), suggesting that an incoming cargo plasmid may not be destroyed by Cas complexes. Nevertheless, a role for the CRISPR/Cas complexes may have been retained as shown in Figure 6, in sRNA profiles, a discrete accumulation of 31 and 42 bp sRNA of very low complexity and a functional Cas6 splicing CRISPR precursor RNA were expressed (Supplementary Table 4, *N. azollae*). CRISPR arrays might not have been recognized if they contained few repeats due to their sheltered lifestyle inside the fern. Rendering *A. filiculoides* and *N. azollae* amenable to genetic manipulation will be crucial to provide evidence for the hypotheses generated by transcript profiling in the future.

## Data Availability Statement

The original contributions presented in the study are publicly available. This data was uploaded at ENA: https://www.ebi.ac.uk/ena/browser/view/PRJEB45214 for the nostoc genomes with the following accession number: PRJEB45214 and https://www.ebi.ac.uk/ena/browser/view/PRJEB45223 for the (s)RNAseq data: PRJEB45223.

## Author Contributions

Fern induction experiments were done by BT and PB, fern crosses and comparisons were done by EG, BT, and unnamed graduate students. The images of shoot apices were generated by VAB, phylogenetic analyses, and miRNA discovery were carried out by LD and NR, sRNA and dual RNA profiling were done by HS. HS conceived the manuscript with all authors contributing to editing of the manuscript.

## Conflict of Interest

The authors declare that the research was conducted in the absence of any commercial or financial relationships that could be construed as a potential conflict of interest.

## Publisher's Note

All claims expressed in this article are solely those of the authors and do not necessarily represent those of their affiliated organizations, or those of the publisher, the editors and the reviewers. Any product that may be evaluated in this article, or claim that may be made by its manufacturer, is not guaranteed or endorsed by the publisher.
