# Supplementary material for: Far-Red Light-Induced Azolla filiculoides Symbiosis Sexual Reproduction: Responsive Transcripts of Symbiont Nostoc azollae Encode Transporters Whilst Those of the Fern Relate to the Angiosperm Floral Transition
Source: Front Plant Sci. 2021 Aug 11;12:693039. doi: 10.3389/fpls.2021.693039 (PMC8386757; doi:10.3389/fpls.2021.693039)
Supplement: Supplementary file 1 [file Data_Sheet_1.PDF]

Supplementary Material

The following Supplementary Material is available for this article:

Table S1 Location and coordinates of *Azolla* collection sites for strains used in the phylogenetic analyses.

Table S2 Red to far-red light intensity ratios and culture densities when testing induction of sporulation by far-red light.

Table S3 Sequence accessions of the fern strains from the phylogenetic trees in Figure. 2A,B. (separate Excell file with sheets for the Trn L-F and Trn G-R).

Table S4 Differentially accumulating transcripts in sporophytes on tube light without (TL) and with far-red LED (FR). (separate Excel file).

Table S5 Read counts of all scored features from *N. azollae*. (separate Excell file).

Table S6 miRNA and target loci transcript abundance in sporophytes in response to FR.

Figure S1 Light quality and fern densities when testing induction of sporulation.

Figure S2 Flow chart to discover conserved and novel miRNA in *A. filiculoides*.

Figure S3 Taxonomy of the *Azolla* strains in this study.

Figure S4 Phylogenetic analysis of the *A. filiculoides* $MIKC^{C}$ responsive to FR.

Figure S5 sRNA sequencing and mapping statistics.

Figure S6 miR172 target sites in AP2/TOE1 transcription factors (eaAP2 lineage) comparing *A. filiculoides* and seed plants.

Figure S7 The miR156a locus in *A. filiculoides*.

Figure S8 The AzfiGAMYB locus Azfi_s0004.g008455 of *A.filiculoides* targeted by miR319.

1 Supplementary Tables

Table S1 Location and coordinates of *Azolla* collection sites for strains used in the phylogenetic analyses.

**Geographic location**	**Coordinates**
Galgenwaard, Utrecht, Netherlands	52°4′35.73″N, 5°8′59.05″E
Nieuwerbrug, Netherlands	52°04'45.5"N 4°48'28.1"E
Hoogwoud, Netherlands	52°43'14.7"N 4°56'25.6"E
Krommerijn, Netherlands	52°05'02.4"N 5°09'32.1"E
Den Bosch, Netherlands	51°41'12.5"N 5°20'17.0"E
Nijmegen, Netherlands	51° 49' 25.1" N 5° 52' 9.501" E
Gran Canaria, Spain	28°04'00.9"N 15°27'38.9"W
Anzali lagoon, Iran	37° 28' 8.007" N 49° 21' 13.208"E
Azolla mexicana Schltdl. & Cham. ex Kunze	IRRI accession ME2001; originally from USA, California, Graylodge, collected by D. Rains in 1978
Azolla microphylla Kaulf.	IRRI accession MI4021; originally from Ecuador, Galapagos, Santa Cruz Island; collected by T. Lumpkin in 1982
Azolla nilotica Mett.	IRRI accession NI5001; originally from Sudan, Kosti; collected by T. Lumpkin in 1982
Azolla caroliniana Willd.	IRRI accession CA3017; originally from Brazil, Rio Grande Sul; collected by I. Watanabe in 1987
Azolla caroliniana	IRRI accession CA3004; originally from Uruguay, Treinta y tres; collected by D. Rains in 1982
Azolla rubra R. Br.	IRRI accession RU6502; originally from Australia, Victoria, collected in 1985

Table S2 Red to far red light intensity ratios and culture densities when testing induction of sporulation by far-red light including the density variation from three replicate cultures maintained for 6 weeks.

Light condition	Red:far-red ratio	Density range of the fern culture [DW g m^{-2}]		
		+/- 50	+/-140	+/- 240
TL	19,13	45 ± 23	139 ± 19	242 ± 18
FR-1	1,16 ± 0,18	49 ± 26	142 ± 22	242 ± 15
FR-2	0,63 ± 0,09	48 ± 25	137 ± 18	239 ± 17
FR-3	0,50 ± 0,06	49 ± 26	140 ± 21	242 ± 19
FR-4*	0,31 ± 0,02	40 ± 16	126 ± 8	221 ± 32

*To achieve the ratio in FR-4, the total PAR was reduced by half.

Table S3 Sequence accessions of the fern strains from the phylogenetic trees in Figure. 2A,B. (separate Excell file with sheets for the Trn L-F and Trn G-R).

Table S4 Differentially accumulating transcripts in sporophytes on tube light without (TL) and with far-red LED (FR). (separate Excel file).

Table S5 Read counts of all scored features from *N. azollae*. (separate Excell file).

Table S6 miRNA and target loci transcript abundance in sporophytes on TL versus FR. miR, miRNA; target locus, MiR target locus; DESeq2 base mean, corrected mean expression computed by DESeq2; log2-fold, log2-fold change comparing the three replicates of ferns exposed to FR versus TL (F vs T); Padj, adjusted P-value according to DESeq2 comparing F vs T; Mercator, annotation of the target locus as predicted by Mercator. Targets with a Padj <0.122 are in bold; those with Padj <0.4.2 are in grey.

	**Target locus**	**DESeq2**	**F vsT**	**F vsT**	
**miR**		base mean	log2FC	Padj	Mercator
**miR156a,b**	Azfi_s0173.g055767	313	0.743	0.355805	SPL-like
	Azfi_s0093.g043231	308	0.775	0.419548	SPL-like
	Azfi_s0048.g030445	275	0.543	0.91417	SPL-like
	Azfi_s0052.g031491	273	0.350	0.95265	SPL-like
	Azfi_s0211.g058143	37	0.251	0.993074	SPL-like
	Azfi_s0068.g036283	22	-0.069	0.996389	SPL-like
	Azfi_s0213.g058306	522	-0.020	0.997365	SPL-like
**miR529a,b,e,f**	Azfi_s0048.g030445	275	0.543	0.91417	SPL-like
	Azfi_s0052.g031491	273	0.350	0.95265	SPL-like
	Azfi_s0211.g058143	37	0.251	0.993074	SPL-like
	Azfi_s0213.g058306	522	-0.020	0.997365	SPL-like
**miR529c,d**	Azfi_s0173.g055767	313	0.743	0.355805	SPL-like
	Azfi_s0052.g031491	273	0.350	0.95265	SPL-like
	Azfi_s0211.g058143	37	0.251	0.993074	SPL-like
	Azfi_s0068.g036283	22	-0.069	0.996389	SPL-like
**miR535**	Azfi_s0093.g043231	308	0.775	0.419548	SPL-like
	Azfi_s0048.g030445	275	0.543	0.91417	SPL-like
	Azfi_s0213.g058306	522	-0.020	0.997365	SPL-like
**miR319**	**Azfi_s0004.g008455**	**103**	**1.930**	**0.002829**	**R2R3-GAMYB**
	**Azfi_s0021.g015882**	**25**	**2.514**	**0.121226**	**R2R3-GAMYB**
	Azfi_s0460.g072042	7	1.699	0.932033	R2R3-GAMYB
	Azfi_s0138.g051134	21	0.433	0.977615	R2R3-MYB
	Azfi_s0041.g026717	44	-0.255	0.984461	R2R3-MYB
**miR172a,d**	Azfi_s0178.g056175	1,319	0.545	0.792255	AP2-like
	Azfi_s0496.g073599	636	-0.281	0.963591	AP2-like
**miR172b,c**	Azfi_s0178.g056175	1,319	0.545	0.792255	AP2-like
	Azfi_s0496.g073599	636	-0.281	0.963591	AP2-like
**miR160a**	Azfi_s0002.g001367	52	0.624	0.859618	ARF
	Azfi_s0019.g015120	243	-0.466	0.900243	ARF
	Azfi_s0099.g044147	633	-0.467	0.914563	ARF
	Azfi_s0013.g013413	377	-0.507	0.940884	ARF
	Azfi_s0697.g082598	168	0.054	0.996389	ARF
**miR160c,d**	Azfi_s0002.g001367	52	0.624	0.859618	ARF
	Azfi_s0019.g015120	243	-0.466	0.900243	ARF
	Azfi_s0099.g044147	633	-0.467	0.914563	ARF
	Azfi_s0013.g013413	377	-0.507	0.940884	ARF
	Azfi_s0697.g082598	168	0.054	0.996389	ARF
**miR171a**	Azfi_s0019.g015002	1,193	-0.225	0.975528	GRAS
	Azfi_s0032.g024755	311	-0.208	0.977615	GRAS
**miR171b**	Azfi_s0019.g015002	1,193	-0.225	0.975528	GRAS

2 Supplementary Figures

Figure S1 Light quality and fern densities when testing induction of sporulation. (**A**) Spectrum of the light under tube light (TL); (**B**), spectrum of the light under TL with far-red LED (FR). Fern densities over 6 weeks of sporocarp induction: cultures refreshed weekly at 41.7 (**C**), 128.8 (**D**) or 208.3 (**E**) g DW m^{-2}. Cultures were weighed and densities re-set at the beginning of each week. Data points are averages of three replicate cultures with standard deviations. (**F**), proportion of mega to microsporocarps over a 9 week induction experiment; (**G**), *AzfiSOC1* mRNA accumulation detected by qRT-PCR in sporophytes grown under TL and FR for 7 weeks.

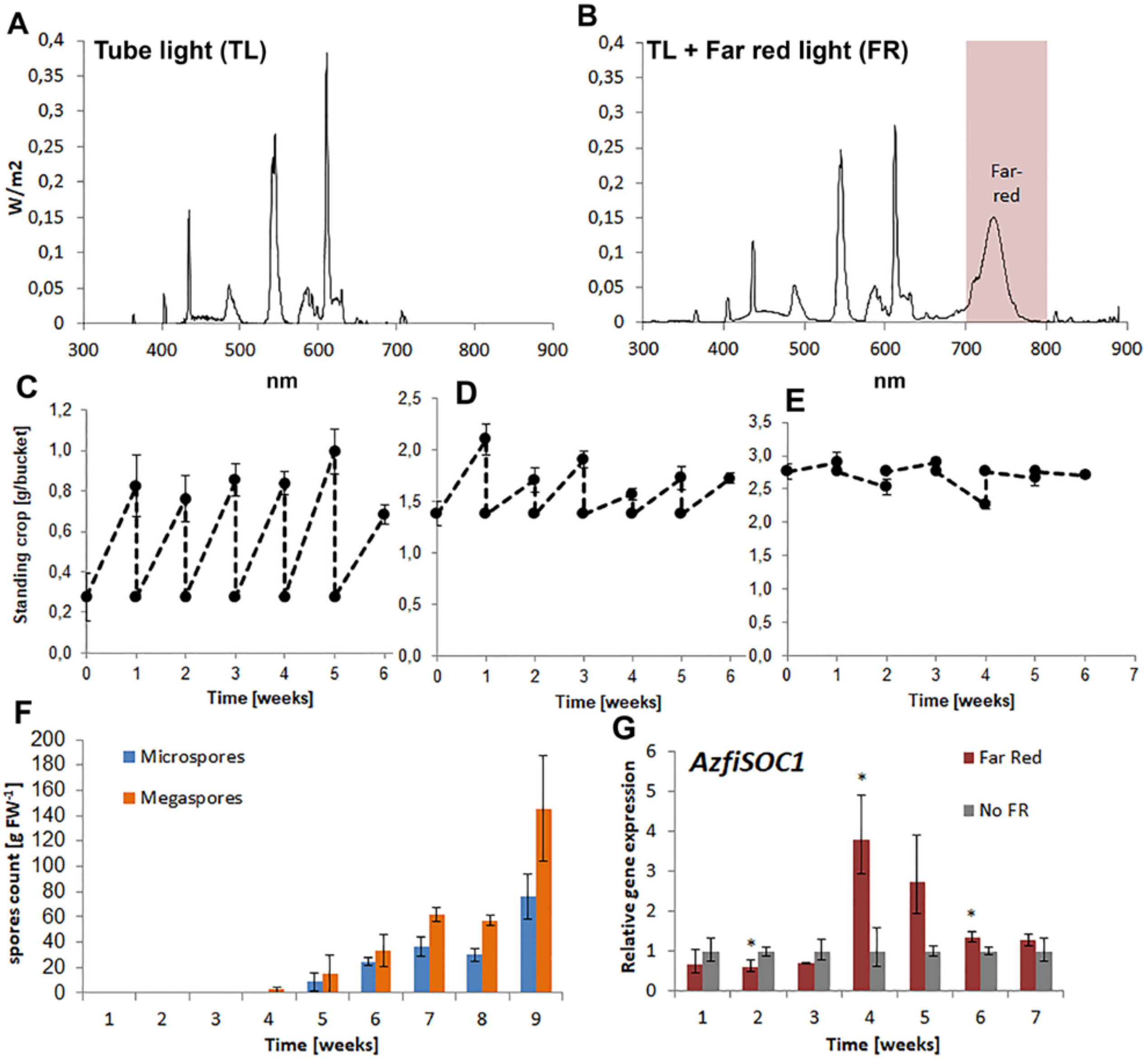

Figure S2 Flow chart of the discovery of conserved and novel miRNA in *A. filiculoides*. miRNA predicted by You et al., 2017 for *A. caroliniana* were used for a Blast search of the *A. filiculoides* genome and hits viewed and further analyzed using IGV (Thorvaldsdóttir et al., 2013). Alternatively, sRNA sequence reads were quality filtered, then collapsed for identical read sequence then only 20-22 nt reads retained for further analyses, the reads were sorted then submitted for analysis with miRDEEP2 and mirDEEP-P2 (Kuang et al., 2018); resulting candidates were verified manually for fold potential in Vienna Fold (Gruber et al., 2008) and expression using IGV, then compared with existing miRNA in miRbase vs 22.1 (Kozomara et al., 2014). Finally, candidate targets were explored using the intersection of results from Targetfinder (Fahlgren & Carrington, 2010) and psRNATarget (Dai *et al.*, 2018).

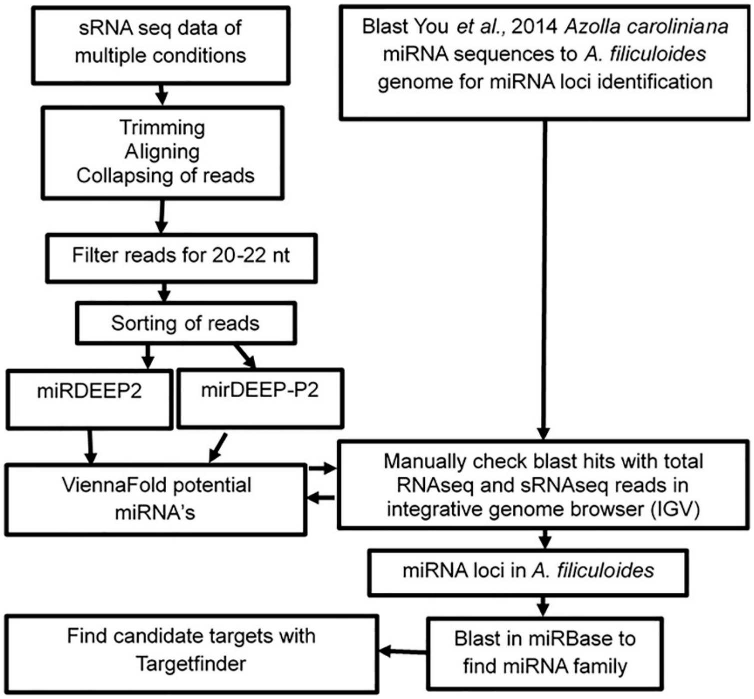

Figure S3 Taxonomy of the *Azolla* strains in this study. (**A**), Sequence variability of ITS1 intergenic regions of rRNA within the genome of each *Azolla* species (Dijkhuizen *et al*., 2018). ITS1 sequences were extracted by alignment to the *A. filiculoides* genome (Li *et al*., 2018). Alignments were visualized using WebLogo: at each position of the ITS, the height of the stack indicates the sequence conservation when compared to the *A. filiculoides* reference, while the height of symbols within the stack indicates the relative frequency of each base within the genome of the species analysed. (**B**), The typical pointed, two-celled, trichomes from the dorsal side of leaves of the Anzali compared to the Galgenwaard strain. Z-stack images were taken of leaves from mature ferns using the Zeiss Axiozoom V.16 binocular with CL9000 LED lights, then reconstituted with Helicon focus software (B, radius 25 smoothing 25).

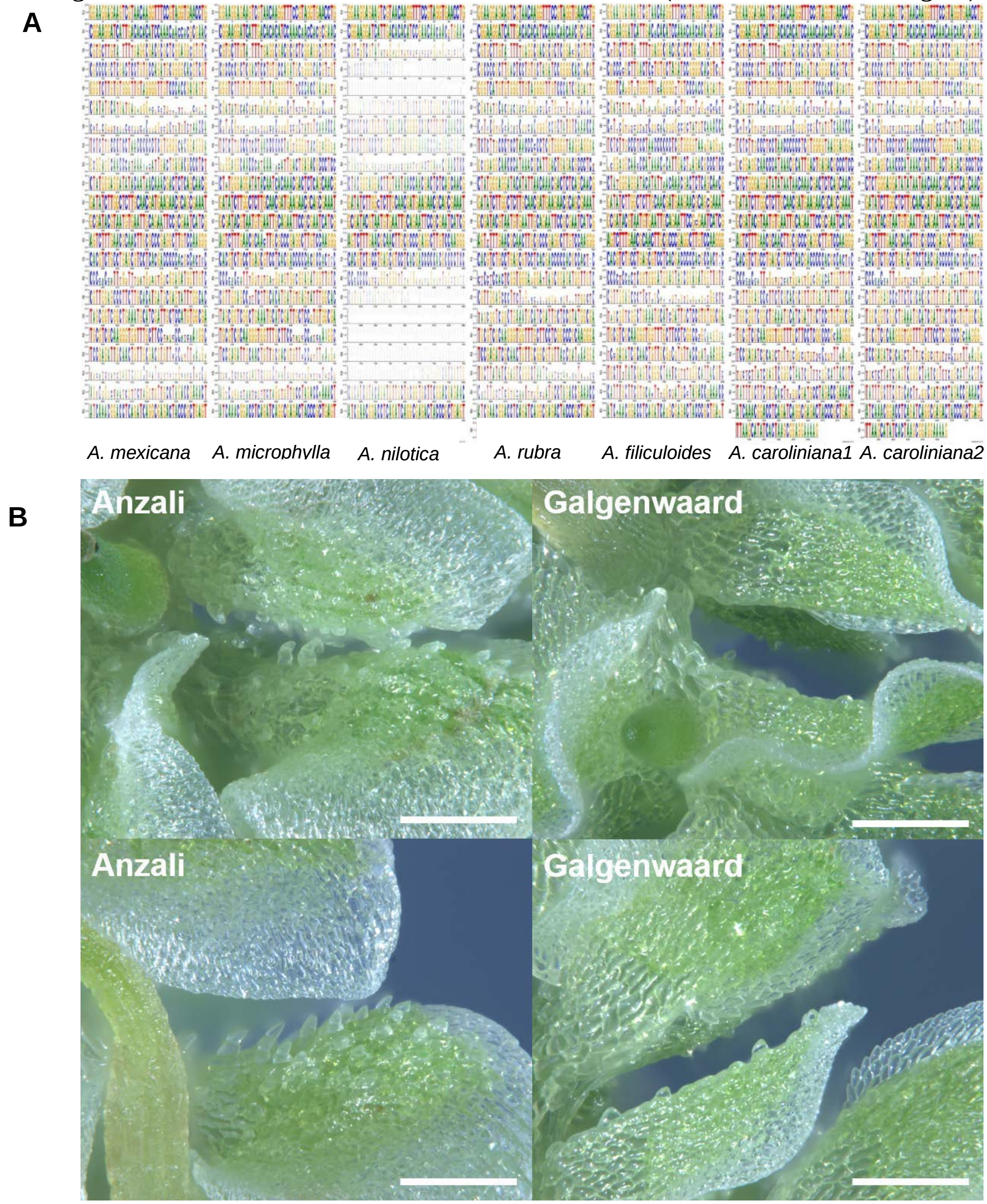

Figure S4. *Azolla* MIKC^C phylogenetic analysis and response to FR. The Azolla MIKC^C gene model encoded by Azfi_s0028.g024032 was annotated manually. Sequences extracted from the genome browsers of each species were aligned with MAFFT E-INS-i (Katoh et al., 2019), then trimmed with trimAl (Capella-Gutiérrez et al., 2009). (**A**), Phylogenetic inferences were computed with IQTREE (Nguyen et al., 2015) and its internal model fitter; 2000 bootstrap determined by SH-aLRT (Kalyaanamoorthy et al., 2017). The tree was rooted using the sequence of *Chara globulosa* MIKC^C (cgMADS1). Nodes with bootstrap support equal or greater than 80% SH-aLRT are indicated. The lower branches of the tree were typically poorly supported by bootstrapping. Branches are color coded as per their plant lineage. Log2 fold-change in response to FR, and Base Mean were calculated by DESeq2 (Love et al., 2014); yellow stars mark significant changes of Padj <0.1. (**B**), draft phylogeny was computed with IQTREE (Nguyen et al., 2015) as in (A), then this draft phylogeny served as a guide for alignment optimization with PRANK (Löytynoja, 2010) of the untrimmed original MAFFT E-INS-i alignment. This optimized alignment was then trimmed again with trimAl and used for inference of the final phylogeny with IQTREE. Bootstrap values are transfer bootstraps calculated with 1000 nonparametric bootstrap trees (Lemoine et al., 201). Transfer bootstrap assays similarity of nodes rather than binary identical or nonidentical nodes in bootstrap trees: it therefore tends to be more informative for bigger trees. All code is deposited on github.com/lauralwd/MIKC_tree. The tree was rooted on a group of algal sequences. Nodes with bootstrap support equal or greater than 50% are indicated. Branches are color coded as per their plant lineage.

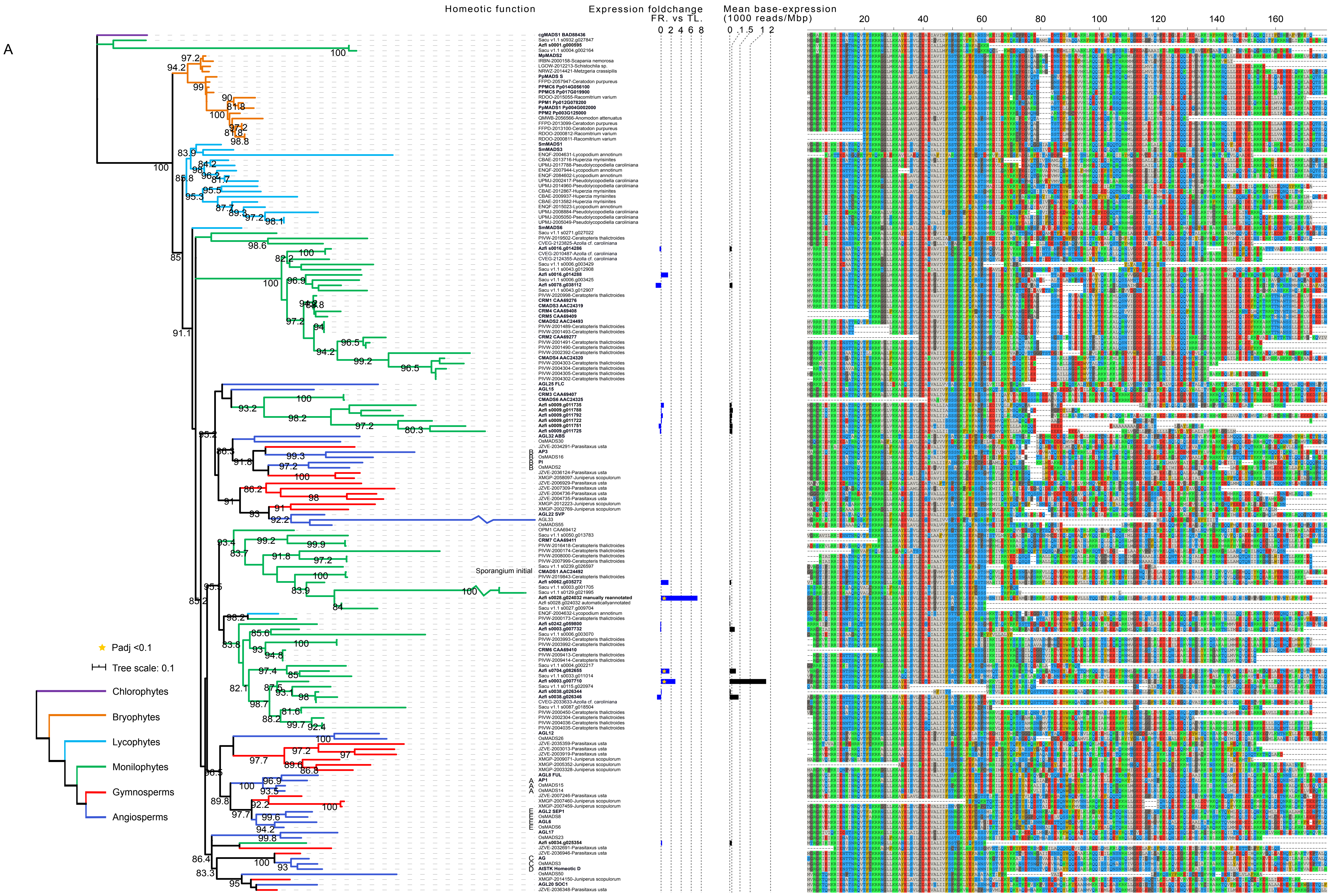
A
Homeotic function
Expression foldchange
FR. vs TL.
0 2 4 6 8
Mean base-expression
(1000 reads/Mbp)
0 .1 .5 1 2
20
40
60
80
100
120
140
160
Sporangium initial
Padj <0.1
Tree scale: 0.1
Chlorophytes
Bryophytes
Lycophytes
Monilophytes
Gymnosperms
Angiosperms

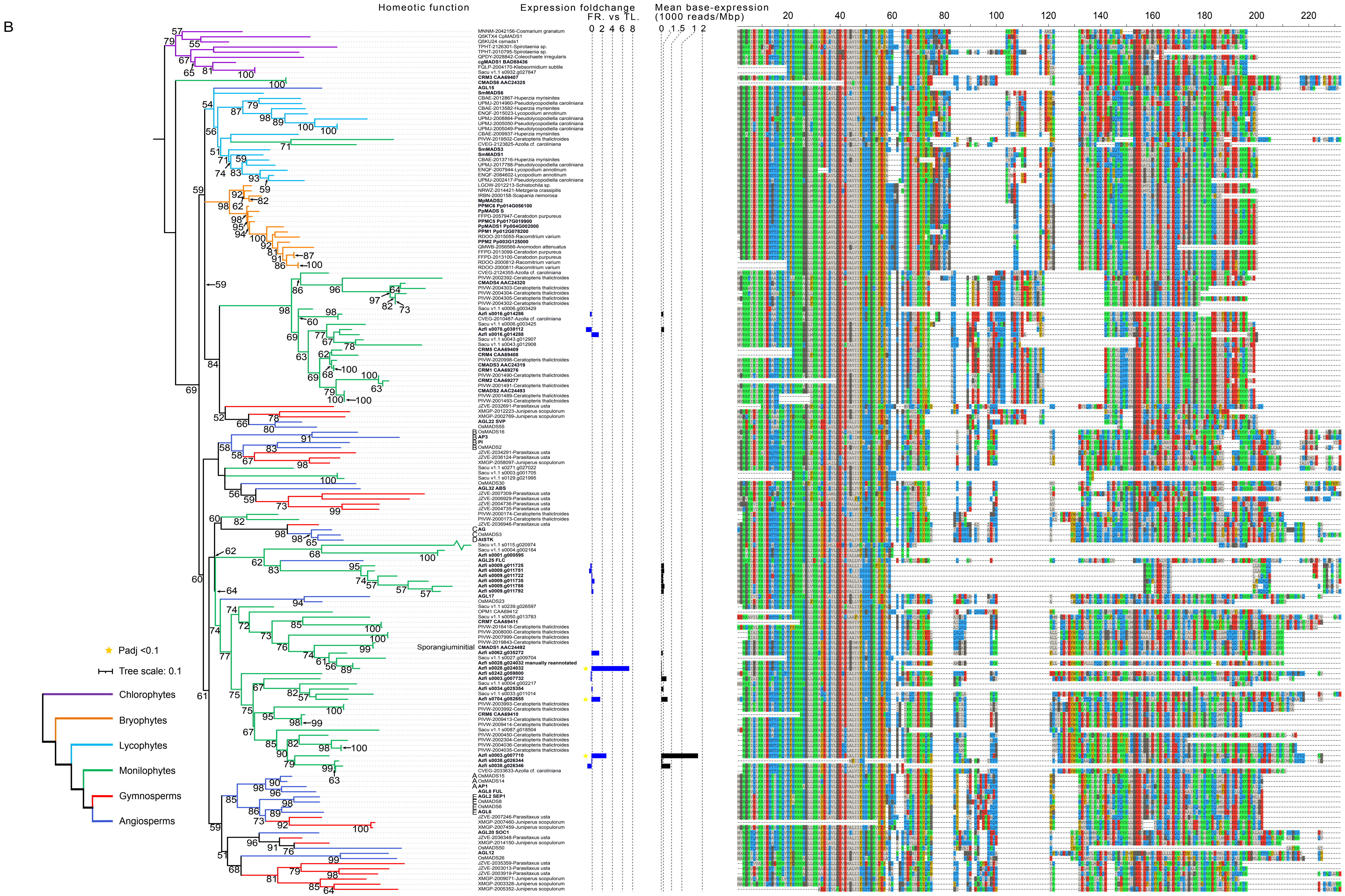
B
Homeotic function
Expression foldchange
FR. vs TL.
Mean base-expression
(1000 reads/Mbp)
Sporangiuminitial
Padj <0.1
Tree scale: 0.1
Chlorophytes
Bryophytes
Lycophytes
Monilophytes
Gymnosperms
Angiosperms

Figure S5 sRNA sequencing and mapping statistics. sRNA was extracted from wild type (wt) sporophytes grown without nitrogen in the growth medium under FR (F1-3) or TL (T1-3); alternatively, extraction was from sporophytes grown with nitrogen under FR as wt (FN1-3) or sporophytes depleted of *N. azollae* (C1-3). (**A**), yield of quality sRNA reads; (**B**) proportion of reads mapping to individual genomes of *A. filiculoides* (Azfi), its chloroplast, *N.azollae* and an associated bacterium from the *Shinella* genus (Shinella) as well as *E.coli* control; (**C**), sRNA mapping on the concatenated genomes of *A. filiculoides* in (B**)**, comparing uniquely mapping versus multi-mappers. (**D**), dispersion when comparing 20-22 nt fern nucleus sRNA in sporophytes on TL with and without far-red LED. (**E)**, DESeq2 on the 20-22 nt from (D), red dots represent sRNA with Padj < 0.1.

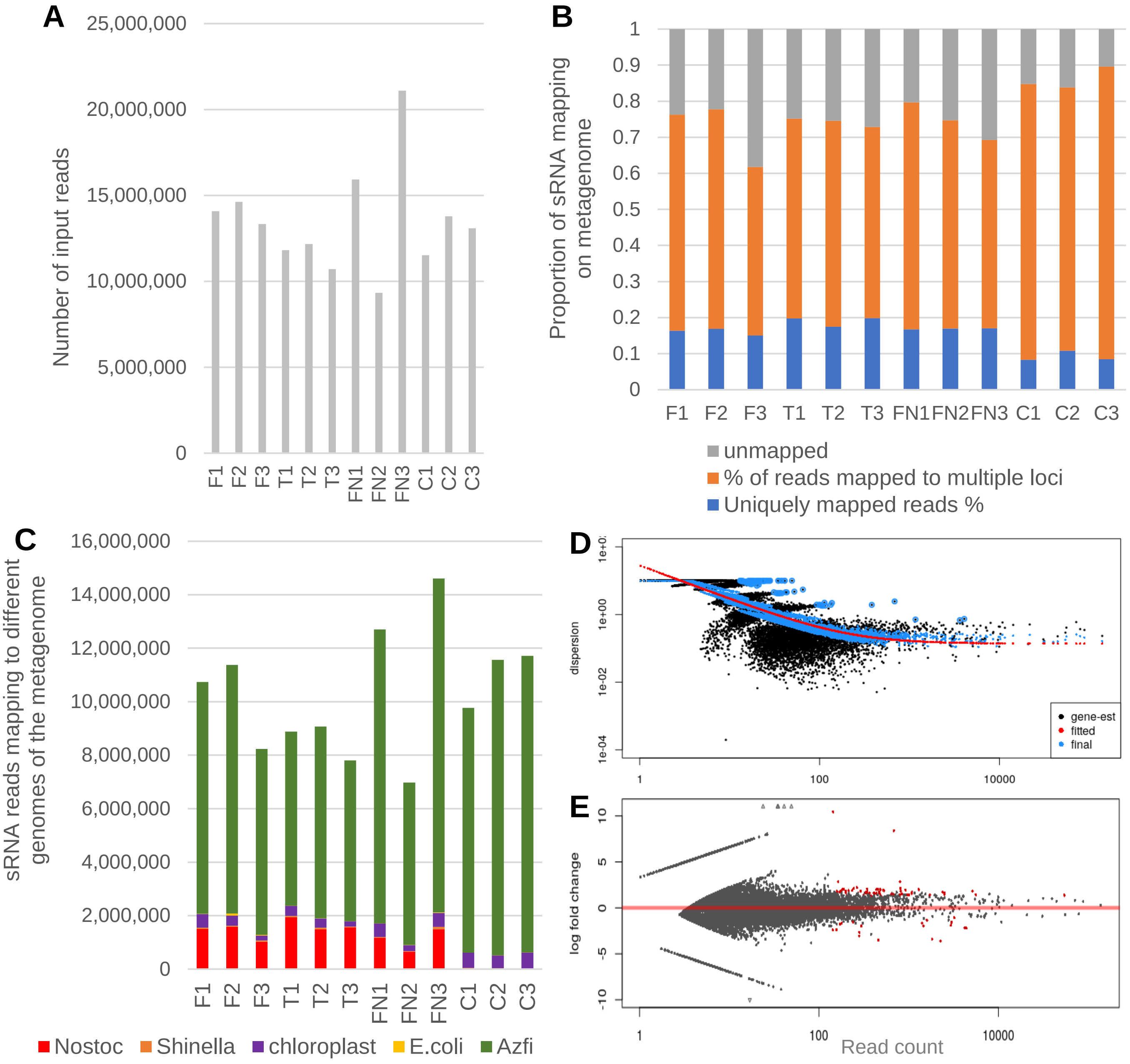

Figure S6 miR172 target sites in AP2/TOE1 transcription factors (eaAP2 lineage) comparing *A. filiculoides* and seed plants.

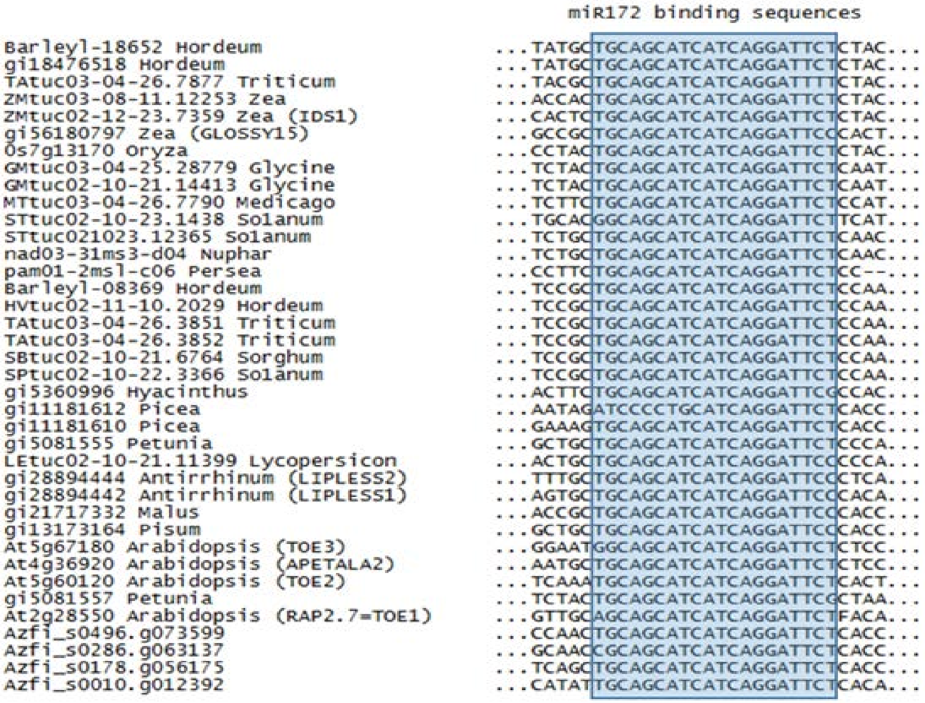

Figure S7 The miR156a locus in *A. filiculoides*. Alignments are visualized using the Integrative Genomics Viewer (Thorvaldsdóttir et al., 2013). (**A**) overview of the locus with the 143 b loop. (**B**) Detail of alignments at the miRNA sequence. (**C**), Detail of alignments at the miRNA* sequence. mRNA enriched RNA-seq reads were pooled from sporophytes grown under various conditions, harvested at different time points in the diel cycle (Brouwer et al., 2017) and are shown under "RNAseq all conditions". Small-(s)RNA reads were from sporophytes grown under different conditions: without *N. azollae* in FR (sRNAseq –*N. azollae*), with *N. azollae* in FR (sRNAseq +FR) and with *N. azollae* in TL (sRNAseq –FR). S, read summary; i, individual reads. Azfi_miR156a (Purple box), miRNA* (Pink box). Amino acid sequences in all three reading frames are shown along with boxed stop (red) and start codons (green). Predicted exon (thick blue bar) from automated annotation at Azfi_s0072.g037014 (Li et al., 2018). (**D**), Azfi_miR156a hairpin folded using Vienna RNAfold (Gruber et al., 2008, predicts -79.74 kcal mol^{-1} released at 22 ^{o}C), miRNA (purple) and miRNA* (pink)

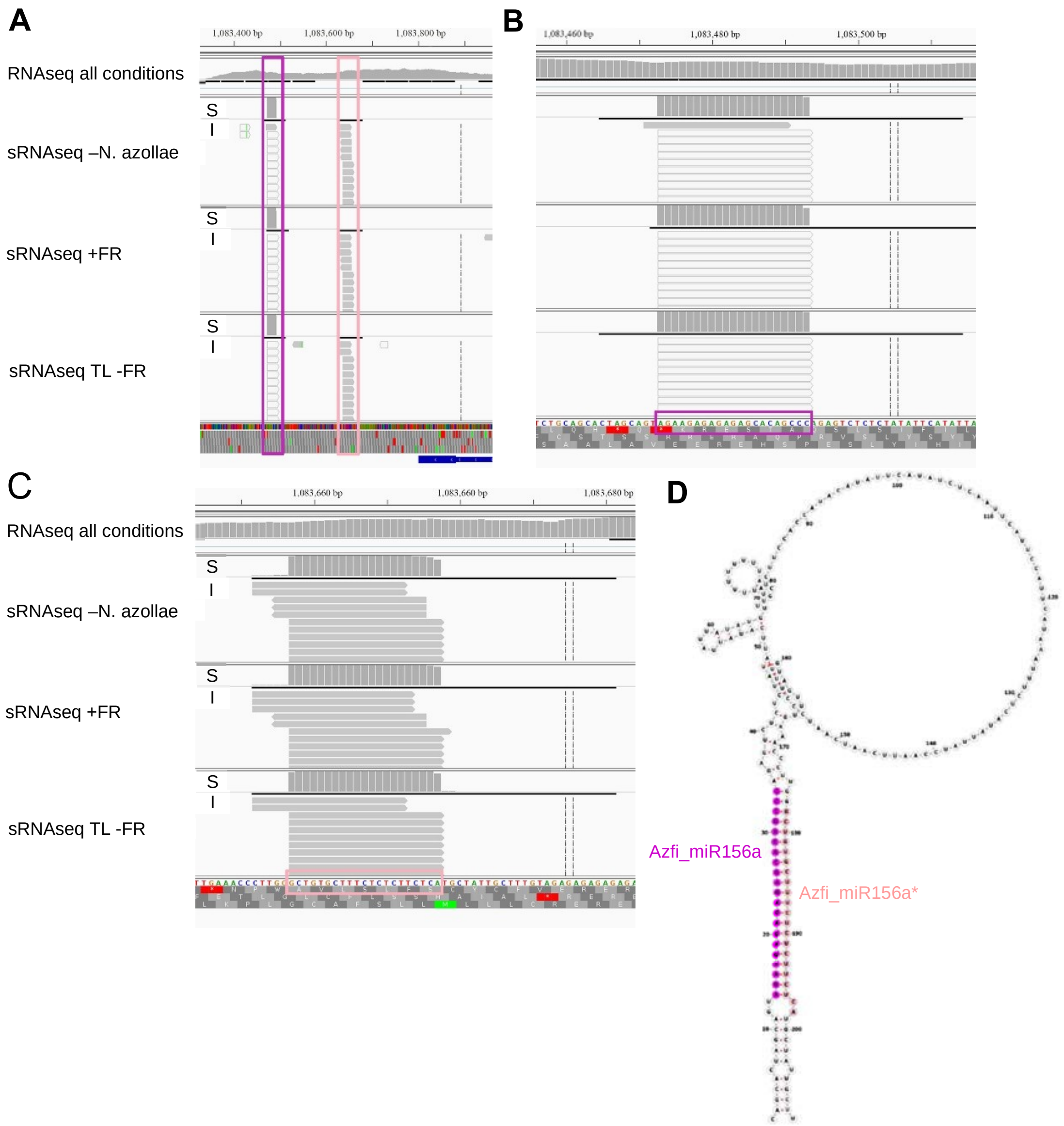

Figure S8 The AzfiGAMYB locus Azfi_s0004.g008455 of *A.filiculoides* targeted by miR319. (**A**) Azfi_miR319 hairpin folded using Vienna RNAfold (Gruber et al., 2008, predicts -145,93 kcal mol^{-1} at 37 ^{o}C); miRNA (purple) and miRNA* (pink). (**B**) Alignment of reads at the locus. (**C**) Detail of miR319 alignment at the miR319 target sequence (red box). S, read summary; I, individual reads. RNAseq all conditions, all reads from Brouwer *et al.*, 2017. Dual RNAseq +FR, reads from sporophytes with FR; Dual RNAseq -FR, reads from sporophytes without FR. Predicted exon (thick blue bar) from automated annotation (Li *et al*., 2018).

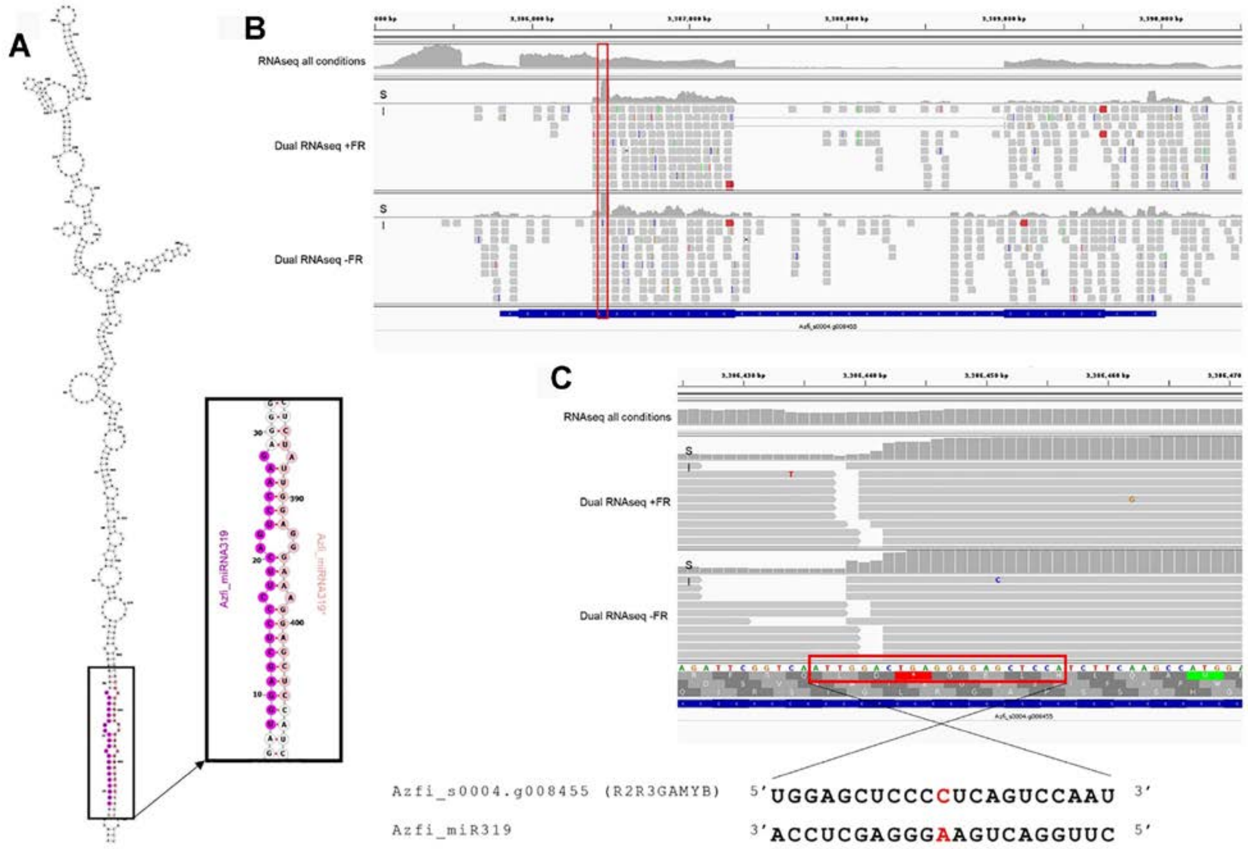

3 References Supplementary Material

Capella-Gutirrez S, Silla-Martnez JM, Gabaldn T. 2019. trimAl: a tool for automated alignment trimming in large-scale phylogenetic analyses. *Bioinformatics* **25**: 1972-3.

Dai X, Zhuang Z, Zhao PX. **2018**. psRNATarget: a plant small RNA target analysis server (217 release). *Nucleic Acids Research* **46**: W49–W54.

Dijkhuizen LW, Brouwer P, Bolhuis H, Reichart G-J, Koppers N, Huettel B, Bolger AM, Li F-W, Cheng S, Liu X, *et al.* 2018. Is there foul play in the leaf pocket? The metagenome of floating fern Azolla reveals endophytes that do not fix N2 but may denitrify. *New Phytologist* **217**: 453-466.

Fahlgren N and Carrington JC. 2011. miRNA target prediction in plants. *Methods in Molecular Biology* **592**: 51– 57.

Gruber AR, Lorenz R, Bernhart SH, Neubock R, Hofacker IL. **2018**. The Vienna RNA Websuite. *Nucleic Acids Research* **36**: W7–W74.

Katoh K, Rozewicki J, Yamada KD. **2019.** MAFFT online service: multiple sequence alignment, interactive sequence choice and visualization. *Briefings in bioinformatics* **2**: 116-6.

Kalyaanamoorthy S, Minh BQ, Wong TK, von Haeseler A, Jermiin LS. 2017. ModelFinder: fast model selection for accurate phylogenetic estimates. *Nature methods*. **14**:587-9.

Kozomara A, Griffiths-Jones S. 2014. miRBase: annotating high confidence microRNAs using deep sequencing data. *Nucleic acids research* **42**: D68-73.

Kuang Z, Wang Y, Li L, Yang X. **2019**. miRDeep-P2: accurate and fast analysis of the microRNA transcriptome in plants (I Birol, Ed.). *Bioinformatics* **35**: 2521–2522.

Leebens-Mack JH, Barker MS, Carpenter EJ, Deyholos MK, Gitzendanner MA, Graham SW, Grosse I, Li Z, Melkonian M, Mirarab S, *et al.* 2019. One thousand plant transcriptomes and the phylogenomics of green plants. *Nature* **574**: 679.

Lemoine F, Domelevo Entfellner JB, Wilkinson E, Correia D, Dávila Felipe M, De Oliveira T, Gascuel O. 2018. Renewing Felsenstein's phylogenetic bootstrap in the era of big data. Nature. doi: 10.1038/ s41586-018-0043-0

Letunic I, Bork P. **2019**. Interactive Tree Of Life (iTOL) v4: recent updates and new developments. *Nucleic acids research* **47**: W256-259.

Li F-W, Brouwer P, Carretero-Paulet L, Cheng S, de Vries J, Delaux P-M, Eily A, Koppers N, Kuo L-Y, Li Z, *et al.* 2018. Fern genomes elucidate land plant evolution and cyanobacterial symbioses. *Nature Plants* **4**: 46-472.

Löytynoja A. **2014.** Phylogeny-aware alignment with PRANK. *Methods Mol Biol.* doi: 10.1007/978-1-62703-646-7_10

Nguyen LT, Schmidt HA, Von Haeseler A, Minh BQ. 2015. IQ-TREE: a fast and effective stochastic algorithm for estimating maximum-likelihood phylogenies. *Molecular biology and evolution* **32**: 268-74.

Slater GS, Birney E. 2005. Automated generation of heuristics for biological sequence comparison. *BMC bioinformatics **6**:31.*

Thorvaldsdóttir H, Robinson JT, Mesirov JP. 2013. Integrative Genomics Viewer (IGV): highperformance genomics data visualization and exploration. *Briefings in bioinformatics*. **14**: 178-92.

Trifinopoulos J, Nguyen LT, von Haeseler A, Minh BQ. **2016**. W-IQ-TREE: a fast online phylogenetic tool for maximum likelihood analysis. *Nucleic acids research* **44**: W232-235.

You C, Cui J, Wang H, Qi X, Kuo LY, Ma H, Gao L, Mo B, Chen X. 2017. Conservation and divergence of small RNA pathways and microRNAs in land plants. *Genome biology* **18**:1-9.
